# A Review of 3D-Printed Medical Devices for Cancer Radiation Therapy

**DOI:** 10.3390/bioengineering13010115

**Published:** 2026-01-19

**Authors:** Radiah Pinckney, Santosh Kumar Parupelli, Peter Sandwall, Sha Chang, Salil Desai

**Affiliations:** 1 Department of Industrial and Systems Engineering, North Carolina A&T State University, Greensboro, NC 27411, USA; rfpinckney@aggies.ncat.edu; 2 College of Engineering, North Carolina A&T State University, Greensboro, NC 27411, USA; sparupel@ncat.edu; 3 Department of Radiation Oncology, OhioHealth, 335 Glessner Ave, Mansfield, OH 44903, USA; peter.sandwall@ohiohealth.com; 4 Department of Radiation Oncology, University of North Carolina School of Medicine, Chapel Hill, NC 27599, USA; sha_chang@med.unc.edu

**Keywords:** three-dimensional printing, cancer, collimator, medical device, radiation therapy

## Abstract

This review explores the transformative role of three-dimensional (3D) printing in radiation therapy for cancer treatment, emphasizing its potential to deliver patient-specific, cost-effective, and sustainable medical devices. The integration of 3D printing enables rapid fabrication of customized boluses, compensators, immobilization devices, and GRID collimators tailored to individual anatomical and clinical requirements. Comparative analysis reveals that additive manufacturing surpasses conventional machining in design flexibility, lead time reduction, and material efficiency, while offering significant cost savings and recyclability benefits. Case studies demonstrate that 3D-printed GRID collimators achieve comparable dosimetric performance to traditional devices, with peak-to-valley dose ratios optimized for spatially fractionated radiation therapy. Furthermore, emerging applications of artificial intelligence (AI) in conjunction with 3D printing promise automated treatment planning, generative device design, and real-time quality assurance, and are paving the way for adaptive and intelligent radiotherapy solutions. Regulatory considerations, including FDA guidelines for additive manufacturing, are discussed to ensure compliance and patient safety. Despite challenges such as material variability, workflow standardization, and large-scale clinical validation, evidence indicates that 3D printing significantly enhances therapeutic precision, reduces toxicity, and improves patient outcomes. This review underscores the synergy between 3D printing and AI-driven innovations as a cornerstone for next-generation radiation oncology, offering a roadmap for clinical adoption and future research.

## 1. Introduction

Cancer is a group of diseases characterized by the uncontrolled growth of abnormal cells spreading across surrounding tissues, making it difficult to treat the body [1,2]. Cancer can develop in any tissue or organ, arising when genetic mutations disrupt normal cell cycle regulation [3]. Additionally, recent cohort studies show that lifestyle factors including diet, exercise, and smoking strongly interact with genetic predisposition to influence the chance of acquiring different types of cancer [4]. These mutations can be caused by various factors, including environmental exposure (e.g., tobacco smoke, radiation, chemicals) [5], genetic predisposition, and lifestyle choices. There are various types of cancer [6], classified based on the tissue or organ where they originate [7], such as carcinoma (epithelial cells), sarcoma (connective tissue), leukemia (blood-forming tissues), lymphoma (immune system), and others. Each type has unique characteristics, progression patterns, and treatment approaches. Extensive reviews explain how the unregulated growth and spreading potential of cancer are caused by genetic and epigenetic factors [8,9,10]. Common signs of cancer include unexplained weight loss, fatigue, persistent pain, or unusual lumps. According to the latest Centers for Disease Control and Prevention report in 2022, of the 1,851,238 new cancer cases reported, 613,349 resulted in death; for every 100,000 people, there were 442.3 new cases and 141.5 deaths, making it the second leading cause of death in the United States [11,12]. Based on the GLOBOCAN 2022 estimates of cancer incidence and mortality generated by the International Agency for Research on Cancer, the American Cancer Society journal offers an update on the global cancer burden. Around 9.7 million cancer deaths (including nonmelanoma skin cancer (NMSC)) and 20.0 million new cancer cases (including NMSC) were reported [13,14]. Further research shows that differences in the incidence of cancer by nation and degree of development are growing; if nothing is done, it is predicted that there will be about 35 million cases and 18.5 million deaths worldwide by 2050 [15]. This highlights the need for continued research to advance medical treatments to mitigate the spread of the disease and protect the patient’s overall health. Treatment options for cancer have evolved significantly, where many different approaches can impact a cancer patient. Advances in precision medicine and genetic profiling have enabled more personalized treatments, improving outcomes for many patients [16,17,18,19]. Next-generation sequencing and biomarker integration are crucial for choosing targeted treatments and minimizing adverse effects, according to precision oncology evaluations [20]. Early detection through screening programs, such as mammograms for breast cancer or colonoscopies for colorectal cancer [21], remain vital for successful intervention. To maximize early cancer diagnosis, customized screening start ages and modalities are supported by the American Cancer Society’s updated screening guidelines [22].

Cancer is one of the major global health challenges. In this review, we discuss how various cancer treatment modalities, including chemotherapy, immunotherapy, surgery, targeted therapy, and radiation therapy, are utilized for cancer treatment. Specifically, this review outlines the various types of radiation therapies, such as photon, electron, and proton techniques, especially using 3D printing processes. Each technique offers unique clinical applications and advantages based on the patient’s requirements, like tumor type, patient-specific needs, and location. A comprehensive overview of how 3D printing has transformed the design and fabrication of radiation therapy medical devices over the past decade is reported, with various use case applications. This review also presents a comparative analysis between traditional manufacturing and 3D printing methods in terms of design complexity, usage, cost, lead time, recyclability/eco-friendliness, dosimetric performance and clinical outcomes. A focused case study on spatially fractionated radiation therapy (SFRT), commonly known as GRID therapy, specialized devices called GRID (a radiation technique that delivers low-dose valleys to protect healthy tissue and high-dose peaks to tumors using a grid that resembles a sieve) pattern collimators are presented for treating bulky tumors. The discussion includes manufacturers, materials used, cost considerations, benefits, and the technical challenges of 3D-printed GRID collimators. Moreover, the limitations and the research gaps of AM in the clinical environment are discussed. Finally, this review concludes with future advances, establishing the Federal Drug Administration (FDA) regulations and integration of artificial intelligence (AI), to enhance the safety and efficiency of cancer treatment techniques.

### Methodology

An evidence-based set of recommendations called Preferred Reporting Items for Systematic Reviews and Meta-Analyses (PRISMA) was created to enhance the clarity, openness, and comprehensiveness of reporting in systematic reviews and meta-analyses. This ensured reproducibility and minimized bias, by offering a standardized checklist and flow diagram to assist researchers in documenting every step of the review process, from literature search and study selection to data synthesis. PRISMA has significance because it improves the validity and reliability of research findings, making them simpler to evaluate and duplicate. Systematic reviews are made more rigorous and informative by adhering to PRISMA criteria, which facilitates improved decision-making in research and clinical settings.

A specific collection of terms was used to query five major databases: Google Scholar, IEEE Xplore, PubMed, Scopus, and Web of Science. They included “3D printing”, “additive manufacturing”, “radiation therapy”, “radiotherapy”, “medical device”, “bolus”, “compensator”, “collimator”, “cancer”, “oncology”, “proton therapy”, “electron therapy”, “photon therapy”, “FDA regulations”, “artificial intelligence”, “GRID therapy”, and “spatially fractionated radiation therapy”. The review focuses on published works from 2015 to 2025 to reflect significant advancements in additive manufacturing and its application in cancer radiotherapy. For this thorough review paper, the full literature search took place over a period of seven months, after which it was compiled into subtopics.

**Figure 1:**
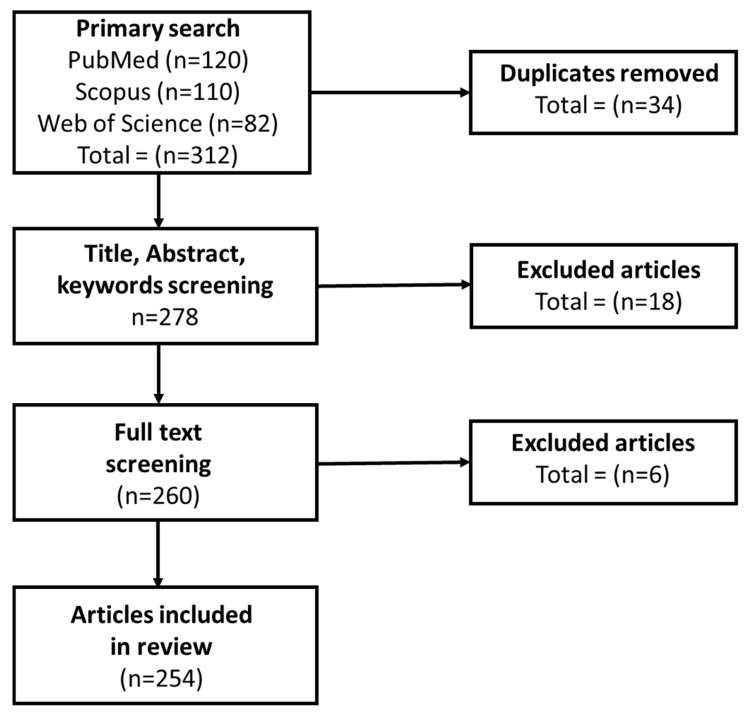
PRIMSA methodology conducted for systematic review.

The PRISMA flow diagram, which summarizes the systematic review procedure, is shown in Figure 1. Every step of the research selection process is shown, including finding records from the designated databases, eliminating duplicates, screening titles and abstracts, determining full-text eligibility, and finally adding studies. This graphic depiction provides objectivity and offers a concise summary of how the original collection of articles was reduced to those that were reviewed.

## 2. Cancer Treatment Therapies

Cancer therapy encompasses a range of treatments aimed at combating the disease and improving patients’ quality of life, affecting both physical and psychological well-being. The main types of cancer treatments include surgery, chemotherapy, immunotherapy, targeted therapy, and radiation therapy. Each of these therapies provides advantages as well as disadvantages, depending upon the type and desired use for the affected cancerous area of the body. While these treatments can be used separately, some cases may involve a combination of these medical cancer treatment techniques.

### 2.1. Surgery

Surgery, one of the most common cancer treatment therapies, involves the physical removal of cancerous tumors, affected tissues, or sometimes entire organs [23]. It is often the primary treatment frequently used for many solid tumors and can be curative in early-stage cancers. Surgery often requires cuts in the skin, through muscles, and sometimes bones, using scalpels, etc. Surgery is considered when cancer is localized and has not metastasized [24]. Additionally, surgery is advised to alleviate discomfort or additional symptoms that are predominantly brought on by tumors. Cryosurgery uses extreme cold produced by liquid nitrogen or argon gas to destroy abnormal tissue [25]. Early-stage skin cancer, retinoblastoma, and precancerous growths on the skin and cervix can all be treated with cryosurgery, also known as cryotherapy. One of surgery’s general drawbacks is that it may harm healthy organs in the vicinity. Moreover, recuperation from surgery can occasionally be prolonged. Bleeding, infection, or anesthesia-related problems can occur after some procedures. Because of this, surgery is not advised for blood malignancies like leukemia [26].

### 2.2. Chemotherapy

Cancer cells can proliferate and divide more quickly than other cells. Chemotherapy, an intensive chemical treatment therapy, is typically another common cancer treatment utilized to treat cancer. It is frequently used with other forms of treatment [23,27]. The two main purposes of chemotherapy are to treat cancer and to alleviate its symptoms. Curing cancer, reducing the likelihood that it will recur, or halting or slowing its growth are all crucial aspects of cancer treatment. It is beneficial to reduce tumors that are causing pain and other issues to alleviate cancer symptoms [28]. Chemotherapy kills cancer cells all over the body with potent medications. It can be given intravenously or orally, and it is frequently given in cycles. Alkylating chemicals, which bind directly to deoxyribonucleic acid (DNA) and prevent DNA replication, are found in certain current chemotherapy medications ultimately causing cancer cell death. Many cancers, including leukemia, lymphoma, multiple myeloma, and sarcoma, are treated with these medications, which act in all stages of the cell cycle. Antimetabolites are another type of chemotherapeutic medication and are tiny substances that serve as fictitious substrates when ribonucleic acid (RNA) or DNA is being synthesized [29]. They do this by replacing the regular building pieces of DNA and RNA, which disrupts the growth of both molecules. Other well-known chemotherapy medications include corticosteroids, mitotic inhibitors, anti-tumor antibiotics [30], and others.

### 2.3. Immunotherapy

In order to combat cancer, cancer biotherapy, also known as immunotherapy, uses the body’s immune system. Immunotherapy fights deadly malignant diseases by modifying the immune system [31]. Treatments include chimeric antigen receptor T-cell therapy and checkpoint inhibitors. Regulatory agencies have approved 17 immunologic products over the last 25 years on the basis of their anticancer activity, either alone or in conjunction with chemotherapy. Levamisole and Bacillus Calmette-Guérin are examples of nonspecific immune stimulants [32]. Another type of treatment used for hormone-sensitive tumors, such as prostate and breast cancer, is hormone therapy, which slows the growth of the cancer by blocking or changing hormone production [33].

### 2.4. Targeted Therapy

Over the past ten years, targeted therapies—such as small molecule inhibitors and monoclonal antibodies—have drastically altered the way cancer is treated [34]. These medications are currently part of the treatment for a number of common diseases, such as multiple myeloma, lymphoma, leukemia, and colorectal, lung, pancreatic, and breast cancer. Compared to conventional cytotoxic chemotherapy, targeted treatments have different toxicities and modes of action including proteinuria, thrombosis, cardiac dysfunction, acneiform rash, and hypertension [35].

### 2.5. Radiation Therapy

Radiation therapy is a form of treatment that utilizes ionizing radiation to shrink cells that are cancerous. Radiation therapy employs high-energy beams to target and destroy cancer cells. It can be delivered externally (external beam radiation) or internally (brachytherapy) [36,37,38]. This type of treatment assists with treating various symptoms and pain relief, targeting a fixed region (tumor) impacted by the cancer. It might also cause harm to the surrounding targeted cells, which could lead to an adverse implication if healthy cells are damaged [36]. With around 50% of all cancer patients undergoing radiation therapy during their illness, radiation therapy is still a crucial part of cancer treatment and accounts for 40% of cancer recovery [39]. High doses of radiation therapy damage the DNA of cancer cells, either by killing them or slowing their growth. When DNA damage to cancer cells becomes irreparable, the cells either cease to divide or die. The body breaks down the damaged cells and eliminates them when they die, as they do not die immediately after radiation treatment. Treatment takes days or weeks before the DNA is sufficiently damaged to cause cancer cells to die. After radiation treatment is finished, cancer cells continue to die for weeks or months [36]. Radiation therapy modalities are commonly classified by the type of radiation employed, such as photons, electrons, or protons, or by the method of delivery.

There are several factors that are taken into consideration to determine which type of radiation treatment should be administered. These include overall health and medical history, the type of cancer you have, the size of the tumor, its location in the body, its proximity to radiation-sensitive normal tissues, whether you will receive other cancer treatments, and other factors like your age and physical characteristics [36]. Regarding the type of treatment, a patient may need multiple cancer treatments such as surgery, chemotherapy, and immunotherapy [36,39]. Radiation can be administered in conjunction with surgery before surgery, during surgery, or after surgery. These measures to assist with the likelihood of the therapy will be effective [36].

As radiotherapy continues to evolve, technology is also helping to assist with further advancements for improvement. One of these advancements that is at the forefront is 3D (three-dimensional) printing of radiation treatment devices. Due to the nature of the patient and their specific needs for care and treatment, 3D printing provides several advantages such as higher precision and lower costs [40,41] for part fabrication. This application is making strides in the medical industry as an effective technique for diagnostics, training, and treatment to become more effective on how to treat cancer with the goal of positively impacting people’s lives.

In summary, Table 1 demonstrates the comparative analysis of the major cancer treatment therapies discussed, highlighting their unique methods of applications, advantages, and potential limitations/adverse actions. By being aware of these choices, patients and medical professionals can make well-informed decisions that are specific to their requirements and the type of cancer they have. Table 1 serves as a valuable reference for evaluating treatment strategies in a comprehensive and patient-centered manner.

The five main cancer treatment modalities (surgery, chemotherapy, immunotherapy, targeted therapy, and radiation therapy) are compared in the table according to their methods, uses, benefits, and drawbacks. This methodical approach provides a comprehensive understanding of the clinical indications, trade-offs, and grasp of each therapy function. The table’s side-by-side presentation of different modalities highlights the variety of cancer-fighting tactics and the reasoning behind multimodal treatment approaches, making it a handy reference for researchers and physicians.

To target cancer cells, each type of therapy uses a different mechanism. Tumors are physically removed during surgery, which works best for early-stage malignancies and confined solid tumors. Chemotherapy is appropriate for systemic cancers like leukemia and lymphoma because it uses cytotoxic drugs to interfere with cell proliferation and DNA/RNA production. Immunotherapy, which has shown promise in lung cancer and melanoma, uses the body’s immune system to recognize and eliminate cancer cells. Targeted therapy provides precise treatment for diseases such as breast and colorectal cancer by focusing on particular molecular pathways. For the treatment of solid tumors and symptom relief, radiation therapy, which employs ionizing radiation to break cancer cell DNA, is frequently used. New methods like 3D printing improve treatment precision.

The table also highlights each modality’s particular advantages. While chemotherapy offers systemic coverage and is frequently used in conjunction with other treatments, surgery can be beneficial for localized disease. In contrast to conventional chemotherapy, targeted therapy minimizes collateral harm through precise targeting, and immunotherapy provides long-term response potential by triggering natural defensive mechanisms. The cornerstone of localized treatment is still radiation therapy, and advancements like patient-specific 3D-printed devices enhance patient comfort and dose compliance. These benefits demonstrate how biological discoveries and technological developments are influencing contemporary oncology to provide more individualized and successful treatments.

Despite its advantages, every therapy has drawbacks that affect how a patient is treated. Surgery is not appropriate for hematologic tumors and carries risks such bleeding, infection, and a lengthy recovery period. Due to the systemic nature of chemotherapy, different medication classes experience different levels of toxicity and side effects. Despite their precision, targeted medicines encounter difficulties such as the emergence of resistance and particular toxicities, including cardiac failure. Even though radiation therapy is very localized, depending on the dose and anatomical location, it might harm nearby healthy tissue and result in delayed adverse effects. These limitations show that in order to minimize dangers and maximize therapeutic benefits, careful patient selection, combination methods, and continuous research are essential.

## 3. Three-Dimensional Printing in Radiation Devices

Three-dimensional printing is an additive process (also known as additive manufacturing (AM) that constructs a 3D part layer-by-layer using a bottom-up approach. Three-dimensional printing technology provides the ability for customized patient models or medical devices specific to the patient’s needs to cater to and address their affected area(s). It provides an effective mode to assist with complex surgery and/or treatment options for planning purposes due to the complexity of design specifications of the medical device, enhancing its fit and function. In turn, treatment planning becomes more effective, and safety is ensured as well.

In the realm of radiation therapy, 3D printing technology has become a ground-breaking modality with enormous potential to enhance patient care and treatment results. Three-dimensional printing has been used in radiation oncology for several purposes, such as creating patient-specific brachytherapy applicators, sophisticated radiotherapy instruments, and customized immobilization devices [42,43]. This technology continues to be utilized through applications that include photon, electron, and proton therapy conducted by these medical devices. Healthcare providers can improve treatment accuracy, minimize side effects, and give patients more comfortable and effective radiation therapy experiences by utilizing the accuracy and adaptability of 3D printing [42,44]. Importantly, this is also a cost-effective solution, with studies revealing promising results in patient care and treatment.

**Figure 2:**
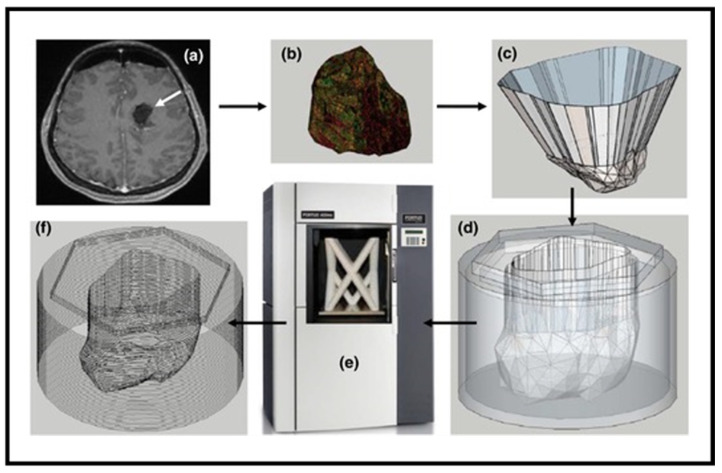
Steps to design and manufacture 3D-printed radiation device. (**a**) CT scan of tumor (Image credit: Dr. Tong Zhu. University of North Carolina, Chapel Hill). (**b**) Extraction of tumor profile. (**c**) Calculation of depth range data. (**d**) CAD (.stl format) of hollow bottle-like bolus; wall thickness tested 0.5 to 3 mm. (**e**) 3D printing—FDM Fortus 400 mc, ABS and PC. (**f**) Clinical integration of the complex bolus design irradiated at 50 Gy via electron beam therapy [41].

Figure 2 [41] illustrates the sequential workflow for designing and fabricating a patient-specific 3D-printed radiation therapy device, emphasizing the integration of imaging, computational modeling, and additive manufacturing. This process begins with Step (a): Tumor Imaging and 3D Scanning, where high-resolution imaging modalities such as computed tomography (CT) or magnetic resonance imaging (MRI) capture the patient’s anatomical details and tumor geometry. These scans provide volumetric data in the form of z-stack images, which are essential for accurate treatment planning. In Step (b): Image Preprocessing, the acquired imaging data undergoes segmentation and conversion into a format compatible with treatment planning systems (TPSs). This step ensures that the tumor contours and surrounding tissues are accurately represented. Depth range calculations are performed to determine the penetration requirements for radiation beams, which is critical for dose optimization. Next, Step (c): CAD Modeling involves creating a digital 3D model of the device using computer-aided design (CAD—SOLIDWORKS 2024 SP5.0) software. The model is typically exported as an STL file, which serves as the standard input for 3D printing. This stage allows clinicians and engineers to incorporate patient-specific anatomical features, ensuring precise fit and effective dose delivery. Step (d): Custom Bolus Design introduces an innovative approach where the bolus—a tissue-equivalent material used to modulate radiation dose—is designed as a hollow structure rather than a conventional solid block. This hollow configuration enables the inclusion of a fillable medium, such as water or other recyclable materials, to adjust radiation attenuation dynamically. By varying the fill material, clinicians can fine-tune the depth–dose profile without redesigning the entire bolus, offering flexibility and sustainability. In Step (e): Additive Manufacturing, the finalized design is fabricated using fused deposition modeling (FDM), a widely adopted 3D printing technique. FDM builds the device layer by layer, utilizing thermoplastic polymers such as acrylonitrile butadiene styrene (ABS), polylactic acid (PLA), or composite blends. These materials are selected for their biocompatibility, mechanical stability, and predictable radiological properties. The layer-by-layer approach ensures geometric accuracy and reproducibility, which are vital for clinical applications. Finally, Step (f): Clinical Integration and Dosimetric Validation involve quality assurance checks to confirm dimensional accuracy and radiation modulation performance. The thin-walled bolus design shown in Figure 2 optimizes electron beam dose distribution by minimizing unwanted scattering and enhancing conformity to the treatment site. This framework is adaptable for photon, electron, and proton therapies, demonstrating its versatility across multiple radiation modalities.

Overall, Figure 2 encapsulates the transformative role of 3D printing in radiation oncology. By enabling rapid prototyping, patient-specific customization, and cost-effective production, this workflow addresses limitations of traditional manufacturing, such as long lead times and poor conformity. Moreover, the ability to incorporate recyclable materials and modular designs aligns with sustainability goals in healthcare. This integrated approach not only improves treatment precision and patient comfort but also sets the stage for future advancements, including AI-driven design optimization and real-time adaptive therapy.

### 3.1. Photon Therapy

Medical devices used for radiation efforts are increasingly being used to administer treatment in various ways. With this form of treatment, there are different types of radiation beams that are used. One of which is photon beams, which can reach deep-seated tumors in the body as little bits of radiation are scattered along its path, oftentimes going beyond the affected area into normal tissue [45]. This form of treatment is called photon beam therapy, where X-ray beams are delivered externally to the body, and this radiation may be slightly stronger [45,46].

#### Photon Therapy 3D-Printed Applications

**Figure 3:**
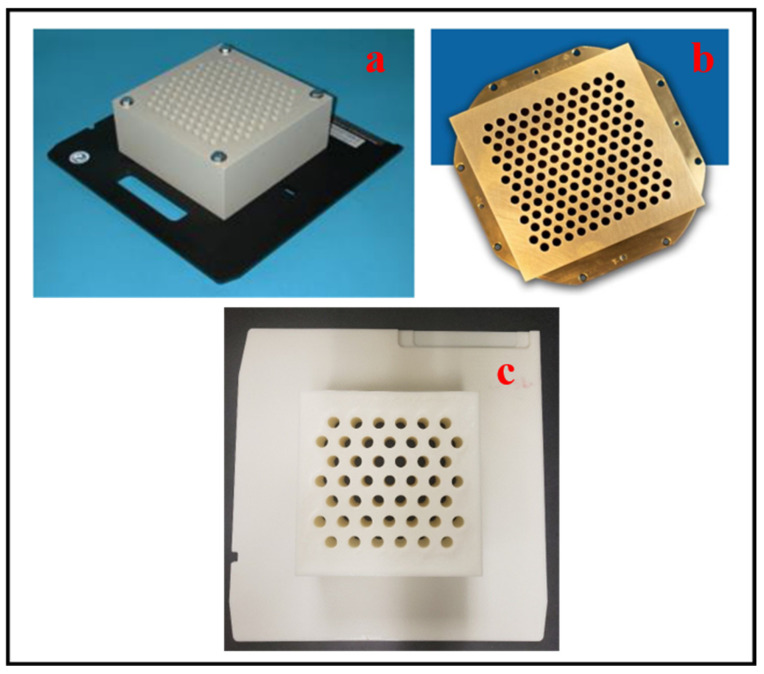
Commercially available GRID collimators. (**a**) High Dose Radiation Grid, Radiation Products Design (7.5 cm, ~10″ × 10″, Cerrobend), (**b**) Commercially available GRID collimator by .decimal Inc.^TM^ (Sanford, FL, USA) (7.5 cm, 7.133 in (x) × by 6.757 in (y), brass, dose-related parameters = 6 MV beam ≥ prescribed dose of 20 Gy, tumors size of 6 to 20 cm diameter at 3 cm depth and at 16 cm diameter at 3, 6, and 10 cm depth, D5–D95 and Equivalent Uniform Dose within ±5%) [48], (**c**) 3D-printed GRID (7.5 cm, 29.5 cm × 29.5 cm, FDM 450 mc printer, ABS).

GRID devices have traditionally been made available through companies who specialize in this market space for radiation oncology. Commercially available blocks or multileaf collimators (MLCs) are known for being the first and most popular device for SFRT implementation [47]. Two of the most commonly used commercially available GRID collimators are manufactured by Radiation Products Design (RPD), located in Albertville, MN, USA, along with .decimal Inc. in Sanford, FL, USA. These two GRID collimators have different dose distributions and peak/valley dose ratios at any given depth due to the various materials (Cerrobend vs. brass) and hole diameters, as shown in Figure 3 below [48].

RPD currently provides two different types of their GRID Photon Block to treat bulky tumors through SFRT [49]. In addition, .decimal manufactures a GRID collimator used for photon therapy [50]. For comparison purposes, Table 2 is provided below to show some key elements for the GRID block compared to a customized 3D-printed model for its formation and consideration [49].

Although the thickness is consistent, the other components are dissimilar, which can play a part or have an impact on treatment. Besides brass, other materials used to fabricate GRIDs for .decimal Inc. include aluminum, blue wax, and silicone [51]. Since the GRID collimator is a standardized tool with physical and geometric parameters, it is ideal for testing as it produces constant dosage heterogeneity qualities [48].

**Figure 4:**
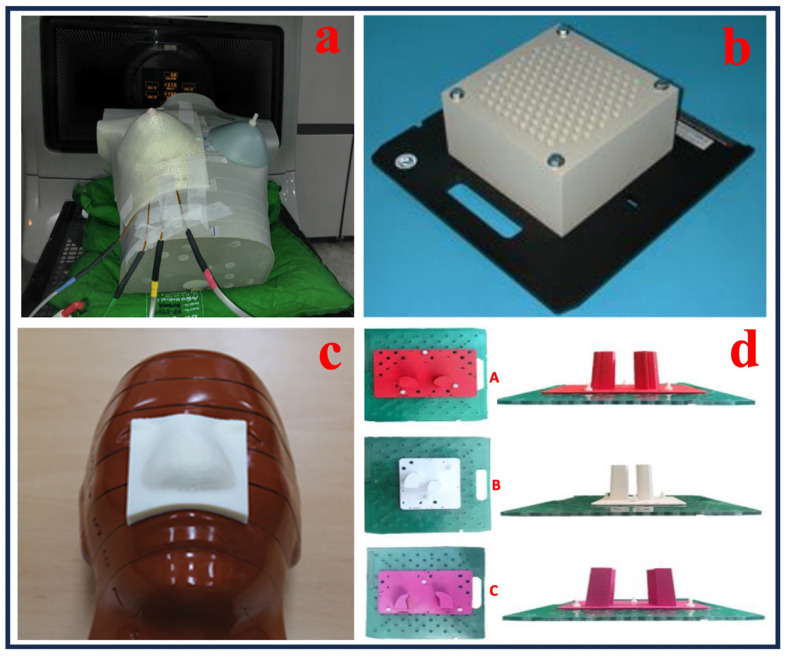
Various 3D-printed medical devices used in proton therapy: (**a**) Bolus (3 mm, 5 mm, PLA, FDM MEISTER 3D BOX Printer, dosimetric parameters = 10 MV, CT scan Philips Brilliance Big Bore, dose-related parameters = HU (Hounsfield unit is a radiodensity scale used in CT imaging where water = 0 HU and air = −1000 HU) = 274, 91.5% (3 mm), and 91.4% (5 mm) [52] © 2016 Creative Commons CC BY 4.0 [52]; (**b**) Commercially available GRID collimators—High Dose Radiation Grid, Radiation Products Design (7.5 cm, ~10″ × 10″, Cerrobend) * [48]; (**c**) 3D-printed customized bolus on RANDO phantom surface (5 mm, LightSpeed RT 16 CT scanner, FDM MakerBot Replicator 2, PLA, dose-related parameters = 6 MV, HU = ~260, passing rate > 90% for 3%/3 mm) © 2014 Creative Commons CC BY 4.0 [53]; (**d**) Top-down view of 3D-printed photon blocks where rows represent patients A, B, C (wall thickness 1.2 mm/8.2 cm maximum depth, FDM Bambu Lab X1 Carbon printer, dose-related parameters = 6 MV, relative dose output/TPS agreement within 2%) © 2024 Creative Commons CC BY 4.0 [54].

Several other devices have also been utilized for use during photon therapy, as shown in Figure 4a–d [48,52,53,54].

Radiation oncology utilizes 3D printing to aid in providing clinical support and serves as an educational aid and mechanism through its applications. According to a systemic review by Rooney et al. [55], over 100 publications within a span of seven years from 2012 to 2019 displayed the most used applications of this 3D printing technology. These include assurance phantoms (26%), brachytherapy applicators (20%), bolus (17%), preclinical animal irradiation (10%), compensators (7%), and immobilization devices (5%). In addition, 71% of those 3D-printed devices utilized photon radiotherapy, followed by electron radiotherapy, then proton radiotherapy. Many of these publication findings represent preclinical studies, whereas clinical applications accounted for two main segments (brachytherapy applicators (48%) and bolus (28%)). The ability of this technology to build these medical devices is probably reflected in this development, attributed to this technology being patient-specific and a low-cost option. Dosimetric evaluation in addition to areas such as print accuracy, cost, and time were the effective reported outcomes. Table 4 of the study shows that 69.9% of the reviewed publications highlighted at least one safety issue or barrier per-taining to 3D printing technology, some of which had a direct bearing on the 3D printing process. (accuracy, cost, print volume) and printing materials (variability in radiological properties, biocompatibility/sterilization, dosimetric variability, hardness impacting patient comfort/tissue simulation, durability/stability). Gugliandolo et al. [56] reported the effect of geometrical and printing parameters on the dosimetry performance of the bolus. The study results demonstrated that 3D-printed boluses provided improved anatomical conformance, improving treatment accuracy, while also performing on a par with conventional boluses. Ricotti et al. [57] leveraged 3D printing as a low-cost option to assess boluses, utilizing this technology with ABS and PLA filament material and assessing various infill percentages. The comparison of a treatment plan with the impact of air inclusion contained by the infill of each bolus was considered. It was also revealed that as the percentage of infill increased, the time to print each bolus was directly proportional, resulting in high density and homogeneity with its material. Consequently, this technology enables altering the position of the build-up area to enhance coverage for the target surface area. A systematic review of 52 studies, conducted by Bochyńska et al. [58], reported that PLA was the most widely used material (57.1%) and that FDM was the most popular printing process (88.1%) for the fabrication of 3D-printed boluses. Although issues like fit accuracy and printing time are still common, the research highlighted better dose compliance and fewer air gaps in comparison to traditional boluses.

**Figure 5:**
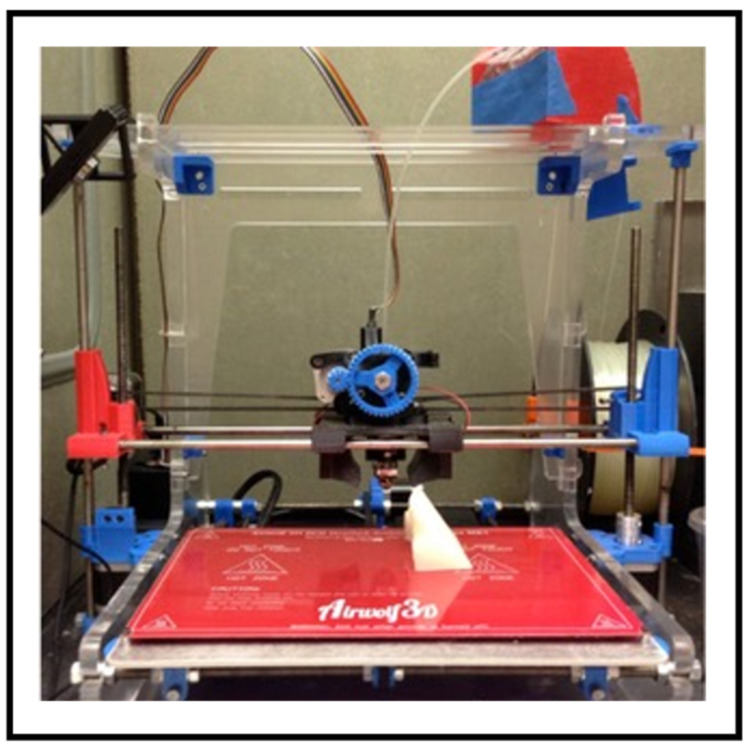
Example of a modified Airwolf printer model, XL 3D, (print area maximum dimensions of 30 cm × 20 cm × 18 cm) used to manufacture a 3D ABS and PLA printed bolus via FDM for external beam therapy treatment; CT scan of Anderson RANDO phantom used [59].

S. Burleson et al. [59] emphasize the viability and quality of producing patient-specific external beam radiation treatment boluses utilizing low-cost 3D printers. This study examines an Eclipse TPS utilizing two printing materials, ABS and PLA, where a gamma analysis was conducted to compare the dose planes. An HU of 260 (material with electron density ratio of 1.14 and mass density ratio of 1.2) was assigned to the printed bolus, which showed how accurately the TPS modeled it [59]. Figure 5 illustrates the 3D-printed bolus using an Airwolf XL 3D-printer [59].

**Figure 6:**
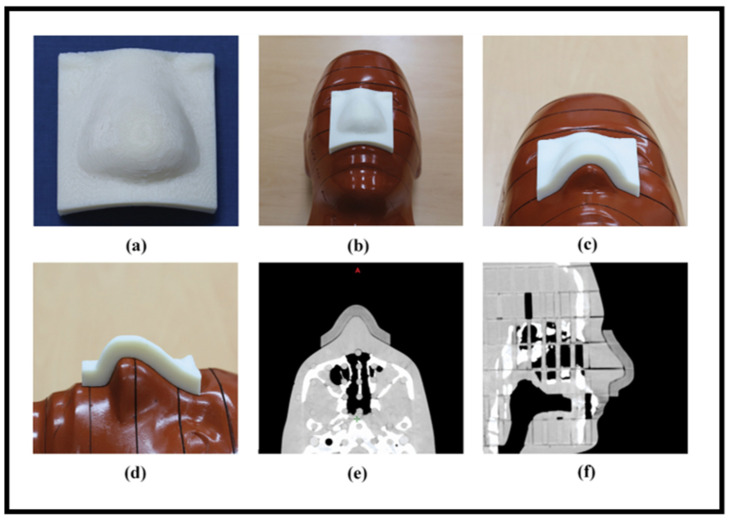
The 3D-printed customized bolus. (**a**) The 3D-printed customized bolus (5 mm thickness, FDM MakerBot Replicator 2, PLA, dose-related parameters = 6 MV, HU = ~260, passing rate > 90% for 3%/3 mm); (**b**) the 3D-printed customized bolus on the surface of the RANDO phantom; (**c**,**d**) cross sectional view of the 3D-printed customized bolus; and (**e**,**f**) axial and sagittal CT images of the 3D-printed customized bolus on the RANDO phantom [53].

Results for the gamma analysis yielded treatment plan accuracy, where 86.5% passed when the gamma criteria included a 5% dosage difference and a 2 mm distance to agreement (DTA); 95% of points passed when the criteria were a 5% dose and a 3 mm DTA [59]. Another investigation evaluated the suitability of leveraging this 3D technology for radiotherapy versus flat boluses that are typically used to aid air gaps due to the lack of formation upon a patient’s skin. The customized 3D bolus printed was comparable to commercially available flat boluses, as demonstrated by its dosimetric measurements for dosage escalation, with the possibility of replacing flat boluses by means of this enhanced methodology, increasing the daily setup’s reproducibility, addressing variable gap issues, and assisting with treatment plan efficacy [53]. Figure 6 illustrates the 3D-printed customized bolus [53].

In these circumstances, the same concept is utilized for radiation therapy. For the first case, a patient has Kimura’s disease involving the auricle, which is a rare inflammatory condition around the ear or earlobe area. Using computed tomography (CT) image reconstruction, a personalized bolus with a thickness of 5 mm was developed. A Dimension 1200 series SST 3D printer was used to produce the bolus. With a maximum air gap of less than 5 mm near the tragus, a repeat CT-based simulation showed that the 3D-printed bolus matched the target location effectively. The 95% isodose line encompassed most of the target region’s surface area [60]. The other instance involved treatment for breast cancer. Air gaps and conformity of the bolus to the breast surface are also discussed as areas of interest affecting dose distribution. In comparison to the commercial Super-Flex bolus used, the 3D-printed PLA bolus administered skin dosage more precisely; average variations between estimated and measured doses with the 3D-printed bolus were insignificant, with a range of −0.7% to −1.1% compared to an anthropomorphic phantom, yet there were notable differences for the dosage with the commercial Super-Flex bolus (−3.2% to −6.3%) [52]. These examples benefited from this technology by improving the target coverage area for irregular surfaces and reducing air gaps as well. Takanen et al. [61] developed 3D-printed boluses packed with ultrasound gel for patients with high-risk breast cancer in a clinical pilot study. Over a 21-month follow-up, the boluses showed good dosage coverage and no toxicity, demonstrating their viability and potential for wider clinical implementation. In addition, this 3D printing method highlights the versatility of material usage, low-cost alternatives and accessibility, an educational realm for training and research, and the quality of these radiotherapy treatments via TPS [62]. Better conformity was executed using PLA and thermoplastic polyether urethane, which also exhibited percentage depth–dose (PDD) measurements—defined as a radiation therapy measurement that shows the proportion of the absorbed dosage at a given depth compared to the dose at a reference depth, usually the point of maximum dose—that were less than 3% different from the RMI457 Solid Water, demonstrating that they could be utilized as bolus materials [63].

### 3.2. Electron Therapy

**Figure 7:**
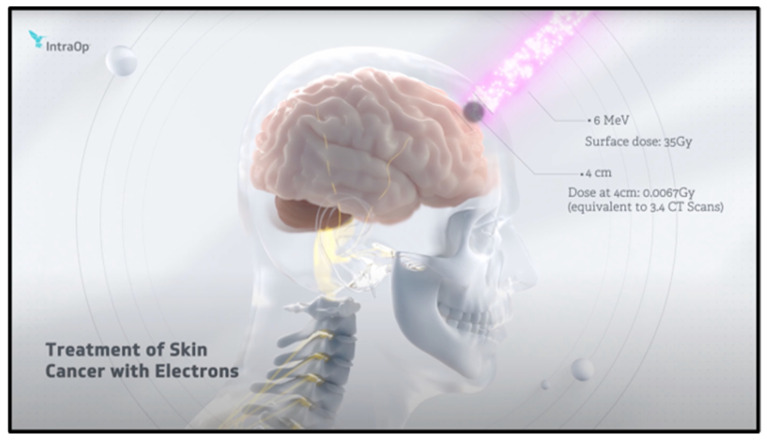
Electron Therapy for Skin Cancer and Dermatology © All rights reserved. IntraOp Medical, Inc. 2025 [66].

Electron therapy, which is also referred to as electron beam radiation therapy, utilizes a beam of electrons with high energy for treating specific types of cancerous tumors. As the electron beam penetrates during the treatment, the electrons targeted on the specific tumor lose energy as they propagate into the skin. It is specifically used for the tumors that are located near the human skin surface because of the inadequate tissue penetration of electrons for deep-positioned tumors [45,64,65]. Some of the common use cases of this therapy include treating superficial tumors, boost therapy, keloids, chest wall irradiation, and periocular and ocular lesions. The advantages include that it is a targeted treatment, causes minimal damage to healthy tissues, treats superficial cancers, and is non-invasive. This therapy can also be utilized in combination with photon therapy for treating tumors with shallow and deep radiation doses, as per the requirement. Figure 7 illustrates how electrons are utilized to treat skin cancer and dermatology through electron therapy [66].

A bolus acts as an auxiliary device that serves as a skin-tissue-equivalent material used in radiation therapy to treat tumors with an enhanced skin dose [67,68]. Commercial boluses available in the market have limitations such as poor conformity, air gaps, setup uncertainties, rigid or inflexible material, and limited reproducibility [69,70]. AM can be utilized to address the above limitations as it enables the fabrication of customized complex bolus structures specific to the patient’s tumor treatment requirements [56,71,72,73,74].

#### Electron Therapy 3D-Printed Applications

**Figure 8:**
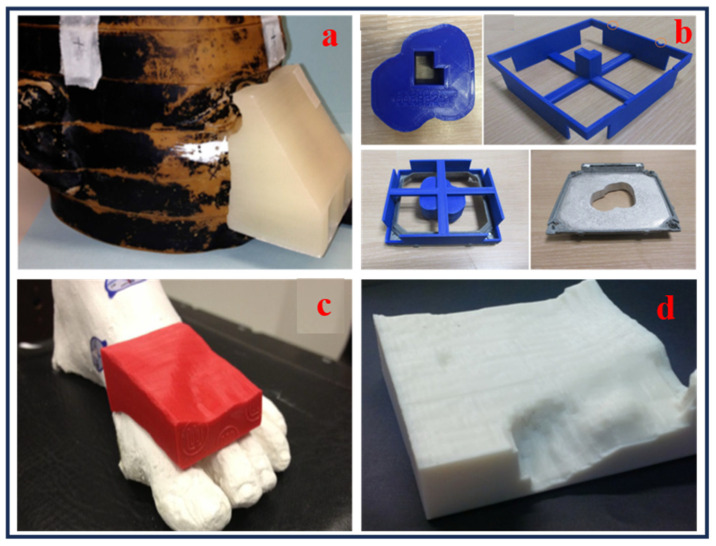
Various 3D-printed medical devices used in electron therapy: (**a**) Printed bolus conformed to the nose of the Alderson RANDO phantom (CT scan, FDM Airwolf XL 3D printer, PLA, dose-related parameters = 6 MV, 9 MeV, and 12 MeV, passing rate 86.5% (5% dose/2 mm) without second CT and 89.6% with second CT) © 2015 Creative Commons CC BY 4.0 [59]; (**b**) 3D-printed patient-specific mold with aperture cut out (6 × 6 cm^2^ to 20 × 20 cm^2^, 2 cm depth using Gafchromic EBT3 film, FDM Raise3D N2 Plus, PLA, dose-related parameters = 12 MeV, 2% output difference between aperture and standard Cerrobend, profile agreement within 1 mm of region) © 2018 Creative Commons-CC-BY 4.0 [76]; (**c**) Foot phantom with bolus added (1 mm thickness, CT scan, FDM MakerBot Replicator 2, PLA, dose-related parameters = 9 MeV, gamma acceptance > 90% (3%/5 mm) © 2014 Creative Commons-CC-BY 4.0 [79]; (**d**) Electron bolus (geometry deviation of design vs. printed 0.84 ± 0.45 mm, CT scan, FDM MakerBot Replicator 2, PLA, dose-related parameters = 9 MeV, HU = 106.5 ± 15.2) © 2015 Creative Commons-CC-BY 4.0 [65].

Compared to traditional manufacturing techniques, 3D printing processes enhance precision and reduce toxicity in radiation therapy by enabling the production of customized electron field-shaping medical devices. It allows for accurate dose distribution, improves patient safety, and accelerates treatment processes [75]. Three-dimensional printing can also be utilized to manufacture patient-specific electron beam aperture cut-outs, customized boluses, dosimetry phantoms, and immobilization devices, without requiring specialized equipment or heavy materials. This advances treatment customization, safety, and workflow efficiency in radiotherapy. Dosimetric verification comparing 3D-printed and traditional Styrofoam molds yielded a 99.9% gamma-index agreement in dose distributions, supporting the integration of 3D printing into patient-specific treatment workflows. However, limitations include a single direct dosimetric comparison and the geometric replication of the mold’s shape [76]. The creation of patient-specific templates and applicators has been made possible by the incorporation of 3D printing into brachytherapy. Rosa et al. [77] examined 74 peer-reviewed research studies on interventional radiation (IRT) this year, with a focus on skin, prostate, and gynecological cancers. Their investigation demonstrated how 3D-printed devices might improve dosimetric accuracy and enable customized treatment plans, particularly in intricate anatomical areas. Additionally, 3D printing has transformed the production of phantoms for imaging quality control and dosimetry. Algethami [78] examined six investigations with 76 patients and found that 3D-printed phantoms and boluses enhanced anatomical accuracy, reproducibility, and dose compliance. Headrests for cancers of the scalp and tongue immobilizers for cancer of the nasopharynx were among the applications. Figure 8 illustrates examples of recent advances in 3D-printed medical devices for electron radiation therapy applications. These include 3D-printed boluses conformed to patient-tailored anatomy (Figure 8a,c,d [59,65,79]) and a custom-tailored mold with an aperture cut-out to meet the treatment requirements (Figure 8b [76]). These examples demonstrate how AM was utilized for manufacturing reproducible, complex designs and patient-specific devices that enhance dose accuracy and overall treatment outcome. Altogether, these findings emphasize the adaptability of 3D printing in improving bolus treatment precision and customization in electron therapy.

Modulated electron radiation therapy (MERT) is a radiation therapy approach that spares underlying healthy tissues while delivering a highly conformal dosage to surface malignancies using modified electron beams. This technique is evaluated where an algorithm was created by the researchers to maximize the distribution of dose for coverage, conformance, and uniformity within the planned target volume. Conformity to both complex anthropomorphic phantoms, along with improved dose compliance, were also favorable outcomes [79]. When compared to a uniform bolus, the MERT plan utilizing a 3D-printed bolus decreased the mean dose reaching the left kidney in the case of the rhabdomyosarcoma patient by 38.2% [79,80]. Zou et al. [65] reported the potential of 3D printing technologies for the fabrication of electron bolus and compensator fabrication in a cost-effective manner. The 3D printing methods such as FDM and selective laser sintering (SLS) and printing materials PLA and polyamide were investigated with CT scans and dosimetric effects. In terms of dosimetric characteristics, FDM with PLA and SLS with polyamide have demonstrated and confirmed the application of proton and compensators fabricated for radiation therapy without any dose deviations. Specifically, the phantom scalp electron bolus fabricated using the FDM method and PLA material on a MakerBot Replicator II printer had a CT HU of 106.5 ± 15.2, yielding a high fabrication accuracy with an average largest deviation of 0.84 ± 0.45 mm from the original design [65].

Misiarz et al. [81] demonstrated the development and application of a novel 3D-printed thin-walled and transparent electron beam applicator for intraoperative electron radiation therapy application. The bolus design was optimized using Monte Carlo simulations, and the measurements were collected based on the International Electrotechnical Commission (IEC) standard recommendations. The measurements of the applicator included applicator diameter (cm), flatness of the off-axis profile (%), dose due to stray X-ray radiation (%), and average dose due to leakage radiation (%). The results of the study illustrated that the proposed applicator shape meets both the radiation leakage protection outside the applicator and the normative standards for forming the radiation field in the beam. Further, these results validate the applicator for clinical applications with biocompatibility, transparency, and a lightweight structure with minimal radiation leakage [81]. Miloichikova et al. [82] reported the utilization of fused filament fabrication 3D-printed polymer materials as beam modifiers to shape electron beam dosage fields in radiation therapy. The viability of this technique was evaluated through a sequence of experiments and simulations using the Monte Carlo method. The results report that by using such polymer materials, the therapeutic electron beams with 6–12 MeV energies can be effectively customized. Further, it enhances treatment efficiency by correcting surface irregularities and addressing tissue inhomogeneities. The dose distribution of the electron beams can be validated by using the developed numerical model even prior to manufacturing for determining compensator geometry for a specific targeted purpose. Tino et al. [83] utilized a multi-material 3D printing method to create anatomically correct phantoms for radiation dosimetry and image quality evaluation. Better quality assurance and dose verification were made possible by these phantoms, especially in complicated therapy situations. To improve accuracy, streamline workflows, and eliminate hazardous chemicals, the study reported by Schulz et al. [84] evaluated the use of 3D printing for customized electron field-shaping blocks in radiation therapy. The study reported the earliest clinical experience report, with an objective to eliminate Cerrobend from an oncology radiation clinic. The methodology of the study involved performing quality assurance (QA), conducting in vivo dosimetry, extracting the cutout aperture from the TPS, and 3D printing the cutout. QA and in vivo optically stimulated luminescence dosimeter measurements were performed (n = 40). The deviation difference was observed to be 4.0 ± 5.2% between the measured skin dosage and the TPS.

A significant application of 3D printing in radiation medical devices is highlighted through the use of 3D-printed plastic compensators in MERT. These compensators optimize electron beam parameters and effectively protect critical organs by modulating depth–dose distribution. Experimental studies focused on optimizing the size and shape of the 3D-printed plastic compensators, demonstrating their effectiveness in shielding essential organs from radiation [80]. Aldawood et al. [41] proposed a novel 3D-printed design and production process for a customized electron bolus device. This thin-walled bolus, when filled with water, optimizes dosage dispersion in electron beam radiation therapy, addressing an unmet need for cancer treatment. Finite element analysis was conducted for dose optimization, while FDM AM facilitated bolus design and production. Materials tested included ABS and polycarbonate (PC), which exhibited the expected radiation modulation properties. The findings illustrate that the proposed thin-walled bolus maximizes electron beam dose distribution, with 3D printing offering an affordable and adaptable solution for radiation devices. However, challenges included unacceptable device deformations during treatment and material degradation after radiation exposure [41]. A retrospective case series reported by Owen et al. [85] highlights the advantages of 3D-printed bolus material in radiotherapy for treating palmar or plantar fibromatosis. Compared to traditional techniques, the use of 3D-printed boluses stabilizes disease progression and reduces patient discomfort. The study employed 3D-printed boluses in radiation therapy using electron and photon techniques, demonstrating their effectiveness in symptom alleviation and improved radiation delivery for fibromatosis [85]. To maximize radiation effectiveness, the study reported by Kaltrine et al. [86] emphasizes the role of 3D-printed customized boluses for maximizing radiation effectiveness. Results of the study indicate that using printed boluses increases the administered dose by 4%. Techniques involved AM of tailored boluses using PLA, demonstrating an increase in treatment dose while maintaining minimal operational costs. The study suggests that patient-specific boluses enhance treatment quality for superficial lesions by increasing skin dose during radiation therapy. The implementation of 3D printing in healthcare settings can reduce bolus production costs while enhancing treatment precision.

Skinner et al. [75] demonstrated a study that illustrates comparative radiation fields and dose transmission assessment between traditional Cerrobend blocks and 3D-printed electron cutouts with tungsten ball bearings. The results of the study reported that 3D-printed cutouts provide precise electron radiation with reduced toxicity, and comparable matching beam profiles and dosage transmission parameters to those of traditional Cerrobend blocks. Identified research gaps include the need for further studies on higher-energy electron beams and advancements in 3D printing technologies. Limitations of the research study include inherent toxicity associated with Cerrobend fabrication and the scarcity of non-toxic, high-electron-density materials.

### 3.3. Proton Therapy

Proton beams have the same ability to reach deep-seated malignancies as photon beams. However, they do not emit radiation while traveling through the body and cease when they reach the tumor, hence these beams may lessen the amount of radiation exposure to healthy tissue [45].

#### Proton Therapy 3D-Printed Applications

Proton therapy is being used progressively more in oncology because of its accurate dose distribution. This enables stronger radiation doses to be administered to tumors while allowing for greater preservation of healthy tissues. According to research studies, numerous types of cancer including pediatric cancers, head and neck cancers, ocular melanomas, and thoracic tumors which include non-small cell lung cancer (NSCLC) benefit from this form of treatment. Clinical studies show that, in comparison to traditional photon-based therapies, proton therapy can result in less toxicity, improved localized disease control, and a boost in a patient’s quality of life. For NSCLC, trials have demonstrated good control and survival rates, and considerable dose reductions in adverse effects such as pneumonitis and esophagitis. In addition, major findings from dosimetric assessments of head, neck, and breast cancers show significant advantages in protecting vital organs including the salivary glands and the heart [87,88,89,90,91].

**Figure 9:**
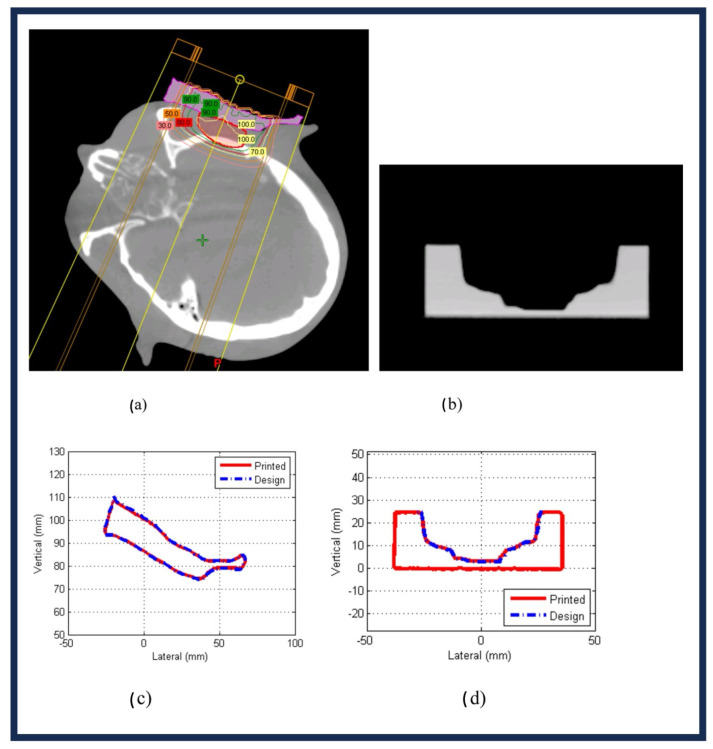
A 3D-printed CT slice of (**a**) the electron bolus (scalp geometry 0.84 ± 0.45 mm, FDM using PLA, dose-related parameters (electron beam, HU: 106.5 ± 15.2) and (**b**) the proton compensator (prostate model 0.40 ± 0.42 mm, SLS using polyamide, dose-related parameters = proton beam, HU: −70.1 ± 8.1; and scanned profiles of (**c**) the electron bolus and (**d**) the proton compensator with the design [65].

As shown in previous radiotherapy types, customization and efficiency are demonstrated by 3D printing techniques to assist with applications catered to the patient. As mentioned for the fabrication of 3D-printed electron boluses, this printing technology is also instrumental in the use of proton compensators. The second method examined was SLS. Polyamide was the material with −70 ± 8.1 HU used to construct the prostrate proton compensator on a commercial EOS 3D printer. The fabrication exhibited excellent precision, with an average largest deviation of 0.40 ± 0.42 mm from the computer-aided design [65]. Scanned CT profile images for both the bolus and compensator in comparison to their respective design files can be viewed below in Figure 9 [65].

**Figure 10:**
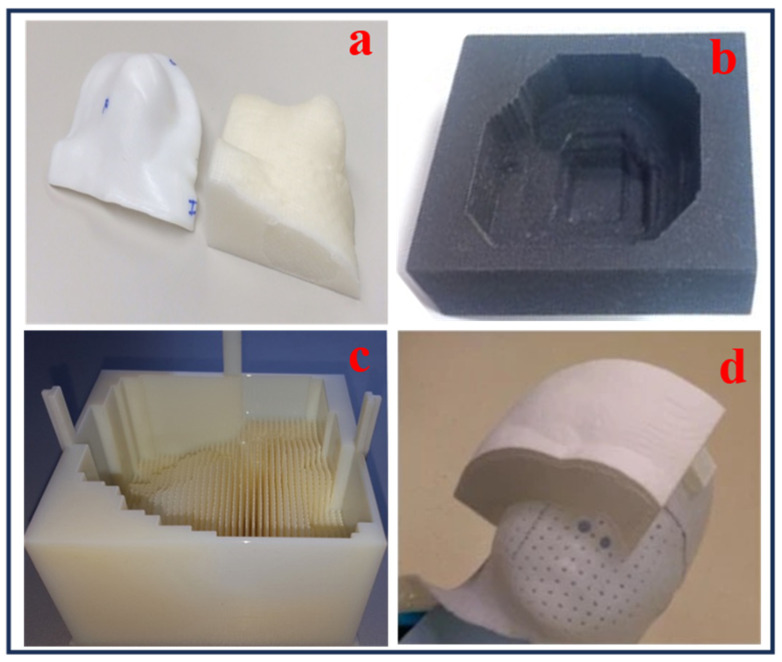
Various 3D-printed medical devices used in proton therapy: (**a**) 3D mold of patient’s scalp and molded Aquaplast RT sheet—left to right (CT scan, FDM Airwolf XL 3D printer, PLA/Aquaplast) © 2015 Creative Commons CC BY 4.0 [59]; (**b**) Proton compensator (geometry deviation of design vs. printed 0.40 ± 0.42 mm, CT scan, SLS EOM printer, polyamide, dose-related parameters = proton beam, HU = −70.1 ± 8.1) © 2015 Creative Commons CC BY 4.0 [65]; (**c**) Lung target (50 mm tall and 3 mm pin base, CT scan, 3D-printed with polymer resin/aluminum, dose-related parameters = 150.68 MeV, irradiated at the synchrotron-based Marburg Ion-Beam Therapy Centre) © 2022 Creative Commons-CC-BY 4.0 [95]; (**d**) Beam compensator (4 cm thickness and ~1kg, Fused Filament Fabrication ATMAT Signal XL printer, PLA, dose-related parameters = 80/100/170 MeV, treatment plan constraints—D_98%_ > 95%, D_2%_ < 107%, prescribed dose—50.4 Gy(RBE) or 55.8 Gy(RBE) in 28 or 31 fractions at 1.8 Gy(RBE)/fraction, water equivalent ratio measurement test—four cubes (1, 2, 4, 6 cm thick) using 130–200 MeV beams) © 2025 Creative Commons-CC-BY 4.0 [97].

Typically, both medical devices are manufactured on milling machines, yet this technology offers the ability for flexibility for customization, treatment efficacy, and the capacity to address clinical guidelines for applications tailored to individual patients and treatment regimens [65]. The exploration of 3D printing for proton radiation therapy has shown promise in advancing proton beam applications. Studies reveal that 3D-printed dosimetric phantoms and immobilization devices can replicate tissue properties suitable for proton radiotherapy when relative water-equivalent thickness values range from 1.0 to 1.3. This capability makes them highly relevant for proton beam therapy [92]. The methodology employed in these studies included experimental calculations, Monte Carlo simulations, and Bragg curve measurements in a water phantom [92,93]. The use of 3D printing technology to create patient-specific radiotherapy equipment has enhanced radiation dosage delivery. This study employed instruments such as compensators and brachytherapy applicators, which assist in producing Cerrobend^®^ grids for spatially modulated proton beam treatments [94]. A notable advancement includes the invention of a 3D-printed range modulator made of polymer resin and aluminum. This modulator improves dose uniformity and conformity for patient-specific cancers in proton therapy. Validation methods for this development included Monte Carlo simulations and experimental measurements, both yielding favorable results [95]. Individualized, 3D-printed whole-body (anthropomorphic) phantoms have also demonstrated their viability for radiation dose measurements, particularly in proton therapy. These phantoms enable precise dosimetry and improve the assessment of radiation exposure from proton beams, which offer the lowest stray radiation exposure among current modalities [96]. Figure 10 illustrates a 3D-printed proton compensator (b) [65], along with other medical devices utilized for proton therapy.

Additionally, 3D-printed proton beam compensators (BCs) have proven effective in lowering out-of-field doses compared to traditional range shifters. This advancement is particularly beneficial for treating shallow pediatric cancers while minimizing secondary radiation exposure. Tools such as radiophotoluminescence detectors monitor dispersed photon beam radiation, while polyallyldiglycol carbonate-based track-etched and thermoluminescent detectors identify secondary neutrons. However, there remains a need for broader demographic studies to investigate long-term impacts on healthy tissues [98]. Using pencil beam scanning (PBS), Wochnik et al. [97] assessed patient-specific 3D-printed proton BCs for treating superficial pediatric cancers. BCs positioned near the patient decreased penumbra (~41–47%) and lateral spot size (~57%) when compared to traditional range changers, enhancing dosage conformity and protecting vital organs. The research reports that 3D printing makes it possible to create accurate, adaptable devices that improve the quality of proton therapy [99] treatment for superficial lesions. Lin et al. [100] examine the application of several range shifters in the treatment of shallow cancers using proton PBS. They evaluate the effects on spot size and dosage distribution of machine-related range shifters (MRSs) and universal patient-related range shifters (UPRSs), such as U-shaped and anterior lateral boluses. Although there are practical drawbacks, including imaging deterioration and collision hazards, the results demonstrate that UPRSs positioned near the patient retain smaller spot sizes and increase dosage compliance. For more widespread clinical use, the authors advise combining automated MRSs with detachable UPRSs for head and neck therapies.

Furthermore, 3D-printed tissue compensators have shown significant potential in optimizing dose delivery in radiotherapy. These compensators enhance dose homogeneity and uniformity while providing tailored patient support. For example, individualized treatment has been demonstrated to benefit patients with Paget’s disease. Preclinical testing on human phantoms compared dosimetric values across different compensators, highlighting the benefits of this approach [101]. A comparison of N-vinylpyrrolidone-based polymer gel dosimetry with 3D proton range measurements revealed range errors of less than 1 mm in anthropomorphic phantoms, underscoring its potential for precise dose administration in clinical proton therapy applications. High agreement was observed between TPSs and Monte Carlo simulations. An MRI scan was used to analyze three-dimensional dose distributions, further supporting the accuracy of polymer gel dosimetry [102]. Lastly, the methodology for creating patient-specific bolus structures using the Eclipse TPS was demonstrated. Custom bolus structures, such as those made from ABSplus-P430 with a relative stopping power of 1.05, enhance treatment delivery efficiency for proton therapy. Results showed no significant dose variation between verification and initial CT scans, validating this novel approach [103].

### 3.4. Performance Evaluation of 3D-Printed Radiation Therapy Medical Devices

This section summarized the significant finding of 3D-printed radiation therapy medical devices over the past decade. While the prior section comprehensively reports the application of 3D printing across photon, electron, and proton treatments, this comparative overview integrates those findings in the tabular form and highlights the key advantages compared to traditional commercial boluses in terms of core performance variables across different radiation therapy modalities. The clinical practicality and dose accuracy of 3D-printed medical devices are generally evaluated using these core performance variables. The core performance variables include medical device type, material used, dosimetry parameters (HU, dose difference, and gamma pass rate), geometric accuracy, cost efficiency, and use case. Table 3 enhances the interpretability of past research and aids future advancements in personalized radiation therapy. This comparative framework is crucial for determining overall trends, evaluating the consistency of reported results, and pinpointing the existing research gaps for the continuous improvement of medical devices in clinical settings.

## 4. Comparative Analysis Between Traditional Manufacturing and 3D Printing for Radiation Devices

Radiation devices have been traditionally fabricated using subtractive manufacturing processes composed of milling, drilling, turning, and a combination thereof. Machining is advantageous for high volume production runs and the use of a variety of materials. However, material wastage can be incurred for complicated geometries and shell-like hollow parts, resulting in higher fabrication costs for custom radiation devices. On the contrary, 3D printing is an additive process which builds parts layer-by-layer with minimal material wastage, and can attain near-net shape geometries without additional post-processing. Three-dimensional printing offers a customized approach with design flexibility to suit specific radiation device technologies. A comparative analysis between traditional manufacturing and 3D printing is elaborated herein.

### 4.1. Design Complexity and Materials

Three-dimensional printing offers greater design flexibility and complexity compared to conventional manufacturing techniques [112]. A key advantage of 3D printing is the ability to create customized or patient-specific medical devices tailored to individual patient anatomy—something that is difficult to achieve through conventional mass-production techniques [113,114]. The ability to rapidly produce and iterate medical device designs is another benefit of 3D printing, as new prototypes can be completed within hours, significantly speeding up the design validation and improvement process compared to traditional methods [43].

Traditional manufacturing imposes design constraints, such as the need for consistent wall thickness and the avoidance of sharp corners, which are required when fabricating electron boluses. In contrast, 3D printing facilitates complex geometries and part consolidation, expanding design possibilities and enabling innovative features for customized phantom devices [115,116]. Thus, 3D printing can be the preferred choice for radiation therapy devices, which allows for greater shape, material, and functional complexities, as well as assembly-free mechanisms and part consolidation, thereby significantly expanding design potential [117].

Material properties represent another key distinction between these two methods. Conventional manufacturing materials are well-established, known for their strength, durability, and predictable performance [118], such as their use for brass and tungsten collimators. While the range of materials available for 3D printing has expanded to include metals, ceramics, polymers, and biocompatible options, 3D-printed materials often exhibit lower strength, heat resistance, and longevity compared to their conventionally produced counterparts [112,118]. A techno-economic comparison of FDM 3D printing and injection molding reveals that AM has significantly lower breakeven values, as traditional manufacturing tends to be more expensive due to the costs associated with mold creation [119]. Despite the higher material performance of traditional methods, 3D printing’s ability to reduce waste, accelerate design iterations, and enable mass customization provides a unique advantage for fabricating radiation therapy devices [112,118].

### 4.2. Cost

Three-dimensional printing has continued to transform biomedical device manufacturing by providing faster production times and more cost-effective solutions. From an economic perspective, FDM equipment is inexpensive, and the process supports the use of various materials in clinical settings [120,121]. This affordability facilitates the experimentation and testing of radiation devices, contributing to the development of optimized prototypes that will be readily field-tested. AM allows for cost-effective customization [120], which is particularly beneficial in creating GRID dosimetry [47], and improves patient comfort during treatment [44].

Three-dimensional printing has demonstrated the capability of cost savings in comparison to computer numerical control (CNC) machining. As 3D printing does not require specialized tools, and each print costs the same, it is less expensive to set up and works well for prototyping, single prints, and small quantities for cancer therapy patients. Because of tooling, programming, and fixturing, CNC machining has greater initial setup costs for devices such as GRID collimators, which makes it less cost-effective for very low-volume runs or quick design modifications [122,123,124].

Additionally, 3D printing presents an opportunity to improve access to advanced medical technology in developing countries, helping to overcome challenges related to design complexity and technical limitations [125].

### 4.3. Lead Time

Traditional manufacturing often requires longer processing times due to the complexity of setup and production, with little to no part variation and minimal customization, which is needed due to patient-to-patient treatment modalities [126]. In contrast, 3D printing allows for greater geometric complexity and customization without the need for specialized equipment or programming, making it faster and more cost-effective for radiation therapy devices [121]. FDM is a material extrusion methodology which presents a low initial cost as an advantage [127], aiding in affordability and accessibility for remote cancer therapy centers. Large-format 3D printing, for example, improves mechanical performance and reduces production time compared to fused filament fabrication, but it can result in lower resolution and poorer surface smoothness [128]. Despite these drawbacks, large-format 3D printing enhances mechanical strength and offers flexibility in material use, including composite materials [128]. Recent innovations include cellulose acetate and cellulose nanocrystals nanocomposite inks developed via solvent exchange postprocessing, offering enhanced thermal and mechanical properties ideal for medical-grade devices [129]. Advances in 3D printing have made it possible to produce patient-specific models and medical implants with improved accuracy and lower costs, enhancing surgical planning and execution [120].

By 2030, the market for AM-generated components is expected to reach $2 trillion, underscoring its potential to reshape industrial production and mass customization, thereby compressing the lead time for radiation therapy devices [130].

### 4.4. Recyclability/Eco Friendly

The concept of being ecofriendly adds a unique dimension to the reuse of materials in both 3D printing and traditional manufacturing for cancer therapy. Three-dimensional printing reduces material waste through precise layer-by-layer production, whereas traditional manufacturing, due to its subtractive nature, often generates substantial amounts of waste and contamination [131]. A reduction in carbon footprint can be achieved by 3D printing’s capacity to create parts closer to radiation therapy centers and minimize transportation. We may infer that AM is beneficial in certain situations since it uses less energy and has quicker manufacturing processes, which reduces waste [127]. A study highlights that PLA prints have a material recovery efficiency of 79.3%, demonstrating the recyclability potential of 3D printing. The recycling of PLA prints reduced the need for new filament by 56%, which contrasts with traditional manufacturing’s limited recycling capabilities and higher plastic waste generation [132].

A circular economy strategy for managing plastic waste through 3D printing emphasizes AM-based distributed recycling as a sustainability solution. By encouraging the involvement of the medical community, streamlining printing processes, and introducing suitable additives, recycled materials could be used for functional components, helping to address the plastic waste crisis and move toward a more sustainable future [133]. Despite the challenges in maintaining material properties over multiple recycling cycles, advances in recycled polymers and bio-based resins have improved the recyclability of 3D-printed products, paving the way for closed-loop systems that reduce dependence on virgin resources [134,135]. This shift highlights the potential of 3D printing not only as a manufacturing method but also as a driver of sustainable material use and waste reduction in clinical settings.

AM enables on-demand customization for patient-specific treatments, offering significant advantages in medical applications [125,136,137]. For example, the reuse of a collimator block for producing GRID dosimetry illustrates the potential for both cost savings and increased flexibility in production [47]. While traditional manufacturing economies of scale make it cost-effective for mass production, 3D printing’s strength lies in producing complex, personalized designs with reduced waste and increased material efficiency. The growing capability of 3D printing to integrate recycled materials and adapt to circular economy models underscores its evolving role in sustainable manufacturing.

### 4.5. Dosimetric Performance and Clinical Outcomes

In traditional manufacturing, several radiation medical devices, such as boluses, compensators, and immobilization devices, are typically fabricated by hand using materials such as wax, gel, or thermoplastic. These techniques are generally accessible and economical; however, they often fall short in terms of personalized anatomical conformance. Especially in complex anatomical locations, air gaps between the device and the patient’s skin might result in impaired target coverage and dosage inhomogeneity. According to findings, traditional boluses might not provide the best surface dosage coverage, which could have a detrimental effect on clinical results and treatment accuracy [56,58,78,138,139,140].

Conversely, 3D printing makes it possible to create highly customized radiation therapy devices that are specific to each patient’s anatomy utilizing CT imaging data. When compared to conventional techniques, 3D-printed boluses and immobilization devices increase dosage compliance, decrease air gaps, and improve consistency, according to recent systematic reviews and clinical trials. For instance, patient-specific phantoms increased the accuracy of dose calculations, while 3D-printed boluses for postmastectomy chest wall irradiation and scalp cancers showed better skin dose accuracy and less toxicity [78,105,141]. Additionally, 3D printing enables intricate anatomical modifications, including tongue immobilization devices for nasopharyngeal cancer that minimize mucosal exposure more effectively than conventional mouthpieces [78].

In summary, 3D printing allows for higher customization and dosimetric precision, that could result in better treatment accuracy and personalized care. Compared to conventional manufacturing, which often limits design complexity due to tooling constraints. Three-dimensional printing enables the creation of complex internal features and lightweight structures, which is particularly valuable for radiation therapy devices [142]. AM builds objects by layering materials, thereby utilizing the majority of materials for device building, whereas subtractive machining results in substantial material wastage, especially for intricate geometries that are sought for electron and proton therapy devices [112,118]. Traditional machining is cost-prohibitive for prototyping and producing customized device designs, as is required for patient-specific cases [143]. Three-dimensional printing addresses this limitation, by providing one-off device designs at a low cost in a variety of polymeric materials [55,144,145,146,147,148,149]. In addition to being a low-cost substitute for conventional material manufacturing techniques, three-dimensional printing can be used to create complex patient-specific devices [55,150,151], helping manufacturers shorten time to market and improve innovation cycles (insert citation). Furthermore, 3D printing may use recycled materials like PLA, ABS, and PETG. G. Prasad et al. [152] highlighted the quality of recycled materials for 3D printing and suggesting sustainable trends for future clinical outcomes. The gains of 3D-printed radiation devices in patient comfort and dosimetric precision imply that additive manufacturing can improve therapeutic results, especially in difficult treatment locations [78,153]. However, before being widely used in radiation oncology, issues including material biocompatibility, cost-effectiveness, and regulatory compliance must be resolved [58,140,154].

## 5. Specific Use Case: GRID Collimators

Spatially fractionated radiation therapy (SFRT), also known as GRID therapy, is a specialized form of radiation treatment that provides ionizing radiation in a non-uniform dose to effectively treat tumors while preserving the surrounding tissue’s normal tolerance [155], a significant interest in GRID therapy for treating large and terminal-stage tumors.

**Figure 11:**
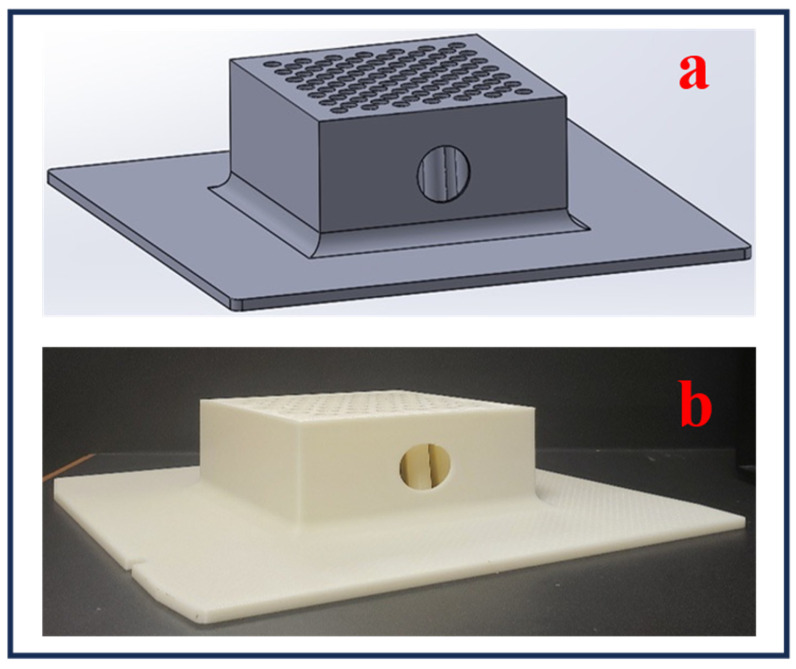
GRID collimator (**a**) 3D CAD model; (**b**) 3D-printed 7.5 cm ABS GRID collimator via FDM Fortus 450 mc printer.

Three-dimensional printing is a preferred fabrication method given its high flexibility for freeform designs and usage of a variety of materials for custom GRID collimator device manufacturing. An example of a 3D-printed GRID collimator is featured in Figure 11. This type of shell GRID design can be filled with Cerrobend or granular tungsten powder to provide targeted pathways for radiation on the tumor sites. Three-dimensional printing permits custom hole designs and can be tailored to specific patient tumor sizes. Moreover, it allows a divergent hole design pattern that follows the divergent path of radiation sources such as LINACs.

### 5.1. Materials for 3D-Printed GRID Collimators

GRID collimators are manufactured from various materials, selected based on characteristics such as density, radiation absorption, and production feasibility. High-density materials that effectively absorb and filter X-rays and gamma rays are commonly used. Lead is the most widely utilized material due to its high density and low cost [156]. For high-energy X-rays, tungsten is favored for its superior radiation absorption capabilities [157,158]. With better radiation screening efficiency than lead, tungsten is increasingly popular [159,160]. Wolfmet tungsten, being over 60% denser than lead, provides enhanced X-ray and gamma radiation attenuation properties [158]. Additionally, aluminum is used for its excellent X-ray absorption capabilities, particularly for lower-energy X-rays [157]. Commercial GRID collimators also employ brass for its strong radiation absorption qualities [161]. Additionally, Cerrobend, a low-melting-point alloy, can be utilized in certain GRID collimator designs [162,163]. Materials for GRID collimator fabrication vary and may include acrylic, blue wax, and stainless steel [51]. Fabrication techniques involve electroplating materials such as copper, nickel, and gold [164]. Research is also being conducted on a new material, tungsten-containing rubber (TCR), for electron grid therapy applications [165]. Material selection depends on factors such as application, radiation energy range, and required image quality. Some grid collimators may combine multiple materials to optimize performance.

In 3D printing applications for radiotherapy, ABS and PLA are the most used materials due to their low failure rates and electron densities similar to water [94]. For 3D-printed GRID collimators, PLA is often used in composite materials containing high-density metals like tungsten. Radiation therapy devices that need to be long-lasting, biocompatible, and structurally sound under clinical settings can benefit from the improved mechanical, thermal, and functional qualities offered by the polymer composite materials used in 3D printing [166]. One example is the PLA-W composite, consisting of 93.1% tungsten powder and 6.9% PLA, which can be 3D-printed using material extrusion techniques and provides effective radiation shielding. Another material, TCR, comprises 90% tungsten by molar composition and is used for electron grid therapy [165]. Additionally, carbon fiber-reinforced thermoplastic polymers offer better thermal stability, making them suitable for high-temperature applications [167]. Other composite materials for radiation shielding include thermoplastics filled with tungsten powder, highly absorbent ceramic powders, and gadolinium oxysulfide [168]. These materials are chosen for their lower toxicity compared to conventional materials like lead and their compatibility with advanced manufacturing techniques such as 3D printing, ensuring effective radiation shielding [161,169]. The mechanical strength and ductility of 3D-printed materials are critical for radiation therapy devices, as they must withstand repeated sterilization cycles and mechanical stress during clinical use; recent advances in nanocomposite formulations have demonstrated enhanced thermal stability, solvent resistance, and corrosion protection, making them ideal candidates for durable and sustainable medical applications [129].

### 5.2. Cost Comparisons

The cost of producing a GRID collimator can vary significantly depending on the materials and techniques used. Customization is crucial in manufacturing these medical devices, and 3D printing has made it possible to customize them at a lower cost [120], improving both affordability and patient comfort during treatment [44]. Table 4 shows a comparison of traditional manufacturing versus 3D printing for GRID collimators.

**Table 4:** Traditional manufacturing v/s 3D printing comparison for GRID collimators.

Criteria	Traditional Manufacturing	Three-Dimensional Printing
Design Flexibility (CAD, G-Code transformation)	Limited flexibility to adapt G-code for over hangs and fine internal features for GRID design.	High level of flexibility for conformal design changes, permitting complex 3D structures.
Material Cost	High volume runs have low material prices; however, subtractive processes produce more waste.	Reduced initial expenses for low volume, customized production and prototyping; material variety and less waste generated
Manufacturing Cost (Machine Cost + Labor Costs)	Higher costs for specialized machining processes, i.e., tooling, drilling, etc., labor; economical for large volume manufacturing	Cost effective for small volume runs and creating prototypes, reduction in labor cost.
Transportation	Increased expenses due to higher volume/large quantities for shipment.	Reduction in cost due to customization, on-site 3D-printed fabrication, and low volume parts.

### 5.3. Benefits of 3D-Printed GRID Collimators

GRID collimators create a peak-to-valley-dose ratio (PVDR) pattern, allowing high doses to be precisely targeted at tumors while sparing adjacent healthy structures [170]. Research has shown that 3D-printed GRID collimators are suited for routine clinical use since they retain their dosimetric performance and structural integrity over time [171]. Rapid prototyping and iteration of designs has enabled previously unachievable treatments, such as neutron scattering of small samples or small field radiotherapy, which require highly specialized or downsized collimator geometries [171,172]. By optimizing dose distribution, enhancing PVDR, and achieving high dose rates, GRID collimators refine spatial fractionation in radiation therapy. Monte Carlo simulations for 3D-printed GRID collimators demonstrated a PVDR of 5 at 10 MV and a mean peak dose rate of 3.06 ± 0.02 Gy/s at 0–1 cm depth [173].

The advancement of AM has expanded its applications across various industries, including healthcare [174]. Previously difficult or impractical to construct complex and highly customizable geometries can now be created using 3D printing processes. Because of its adaptability and flexibility, medical professionals can modify the collimator design to meet clinical requirements, which enhances performance and opens new avenues for research and therapy [171]. Customization plays a critical role in manufacturing medical devices, and AM facilitates the production of tailored devices at reduced costs [120], enhancing patient comfort during treatment [44]. With 3D printing, collimators may be made in-house at a fraction of the time and expense of traditional machining. For instance, collimators may be made for around $30 in a single day, while traditional fabrication methods are expensive ($1000) and require longer lead times (2 to 3 weeks) [171,172]. Moreover, 3D printing streamlines workflow in healthcare settings by reducing human labor and the time needed to construct collimators [54,175]. Because 3D printing can result in significant material and manufacturing savings, it is a financially viable choice for healthcare facilities [176]. The ability to translate specific medical requirements into precise physical components for clinical use presents a significant opportunity for improved treatment outcomes. Accurate dose modulation and spatial resolution in radiotherapy and imaging depend on the manufacture of complex grid patterns and small-scale features, which the AM technique enables to be highly precise and repeatable [171,177]. High-density, radiation-attenuating materials, such as tungsten or boron carbide, can be employed in advanced 3D printing procedures to provide beam shaping and effective shielding that is on par with conventional metal collimators [158,171]. Research indicates that 3D-printed photon blocks have dosimetric characteristics that are similar to those of conventional materials, with negligible variations in dose output [54]. Wolfmet 3D’s process allows for the creation of highly intricate component designs and geometries unattainable through conventional milling and turning [158]. Additionally, denser septa result in reduced image dispersion, yielding more precise imaging compared to traditional lead collimators.

In addition, 3D-printed collimators that use non-toxic materials improve patient safety by lowering the risk that comes with using more conventional materials like Cerrobend [54]. The versatility of the 3D-printed GRID block enables its use with both 6 MV and 18 MV photon energies, each with distinct dosimetric properties [178]. For radiation safety and biocompatibility to be confirmed, material selection is crucial. The way a material interacts with radiation and live tissue depends on its properties, including its atomic number, chemical composition, and additives. Lead and other heavy metal shielding work well; however, there are key issues with their toxicity [179,180,181,182]. As a result, there has been a shift toward polymer-based composites that are rich in low-Z elements (such as carbon and hydrogen), which provide flexibility, are less poisonous, and more closely match biological tissues [179,180,181,183]. These polymers can achieve better radiation shielding while remaining compatible with biological systems by adding certain fillers and fine-tuning additives, which will lessen negative effects and make regulatory compliance easier [179,180,181,182,183]. Also, material stability under exposure is essential in high-radiation environments. Materials must be resistant to oxidation and depolymerization, two chemical breakdown processes that can weaken structural integrity or release toxic chemicals. This is particularly important when sterilizing materials, which is frequently performed using radiation and requires them to endure exposure without leaking contaminants. Because of their dependability, durability under sterilizing conditions, and proven safety, titanium, and several high-performance polymers (such as PEEK or medical-grade silicones) are favored. The best option depends on the device application and the expected radiation exposure, necessitating careful consideration to guarantee long-term, safe operation [180,184,185].

### 5.4. Challenges of 3D-Printed GRID Collimators

Despite offering unique therapeutic benefits in radiation therapy, 3D-printed GRID collimators face limitations that hinder their widespread adoption and efficient operation. Challenges related to dosimetry, treatment planning, and integration with existing systems arise due to the spatially fractionated radiation doses [48,186]. Many TPSs struggle to accurately compute and simulate the complex dose distribution produced by GRID collimators, leading to dosimetric uncertainties [48]. Additionally, the spatial modulation of the target volume deviates from conventional radiation procedures, which typically aim for uniform dose distribution [186]. As a result, existing protocols and QA methods must be carefully evaluated and adapted to accommodate 3D printing paradigm shift. Prior to patient usage, each 3D-printed device must undergo a rigorous, frequently time-consuming QA process to ensure that it satisfies clinical standards [84].

Even with advancements in 3D printing that facilitate the production of customized GRID collimators, implementation challenges persist. These include material selection [187,188,189,190,191], equipment accuracy, cost [55], and safety concerns inherent to this treatment approach [55]. Materials used to build collimators must be biocompatible and capable of efficiently attenuating radiation. Many materials used in 3D printing are not adequately shielded from radiation or may deteriorate over time when exposed to ionizing radiation. Traditionally, these devices have been manufactured using Cerrobend, brass, and, in some cases, lead or tungsten. Compared to conventional metals like brass, 3D-printed plastics, even metal-filled filaments, frequently need to be thicker to provide the same level of shielding, which can lead to bulkier devices and higher surface doses. Also, there is a supply chain risk for clinical operations, since some specialized 3D printing filaments and powders may not be readily available or consistent. Furthermore, initial capital expenses include purchasing the appropriate 3D printers and supplies, as well as equipment maintenance and employee competency [175].

To alleviate these challenges, custom configurations can be implemented to accommodate specific PVDR in 3D-printed GRID collimators. These can be attained by varying several design and material parameters. On the design front, this includes manipulating the depth of the GRID collimator, the radiation pathways (holes), and the center-to-center distances between holes. Material types such as Cerrobend or tungsten granules can be filled within 3D-printed GRID collimators to control the radiation attenuation levels to suit the application intent. Optimization studies need to be conducted to evaluate the effect of several input parameters including hole geometries and material densities on the dosimetry output of GRID collimators.

#### 5.4.1. Limitations of Additive Manufacturing in Clinical Environments: Calibration, Traceability, Sterilization Protocols, and Cross-Institutional Reproducibility

Additive manufacturing (AM) enables patient-specific device fabrication; however, its clinical deployment remains constrained by persistent limitations in 3D printer calibration and process control, material traceability, biocompatibility documentation, and cross-institutional reproducibility. The U.S. FDA’s guidance Technical Considerations for Additive Manufactured Medical Devices highlights device design, software workflow, material controls, process validation, and sterilization as key risk areas; it also underscores that many AM parameters can affect performance and must be validated and documented [192].

##### Calibration Effect on Geometric Accuracy

The accuracy of 3D-printed parts is sensitive to layer thickness, build orientation, bed temperatures, filament rheology, file conversions, and environmental conditions. Regulatory agencies such as the FDA require the evaluation of parameter inter-relationships, monitor processes, and define worst-case builds for verification. Standards efforts emphasize machine acceptance testing, operator qualification, and verification of AM-specific test artifacts, but coverage varies by modality and clinical use [193]. Quality-assurance studies further demonstrate that segmentation and print modality each contribute to measurable dimensional error, requiring end-to-end verification rather than relying on device specifications alone [194,195]. Calibration does not end at the build: cleaning, UV cure/anneal, and sterilization can deform parts. Recent in vitro and clinical-adjacent studies show that steam sterilization can alter the geometric accuracy of 3D-printed surgical equipment, with material- and protocol-dependent outcomes [196]. A comparative study across extrusion materials found severe deformation after autoclave for several polymers, underscoring the need to validate sterilization “as-processed” for each 3D-printed device [197,198].

##### Material Traceability and Biocompatibility

The 3D-printed devices to be used in clinical settings require that documentation must link the finished device to the specific lot of feedstocks, processing history (e.g., resin reuse ratio, drying logs), post-processing parameters, and sterilization/cleaning cycles. This aligns with ISO 13485-style quality systems and the FDA’s expectations for material controls and labeling. Manufacturers and some AM suppliers now operate ISO 13485-certified QMS to support medical applications [199], but hospital PoC labs frequently lack formalized supply-chain controls and regrind/resin reuse specifications [200,201,202,203]. Importantly, resin/filament vendors emphasize that biocompatibility claims are held only when users follow a validated workflow (printer, settings, wash/cure), because processing alters the final chemistry, and deviations may void biocompatibility. Without re-validating the wash/UV parameters, extractables/leachables exceed cytotoxicity thresholds in verification testing, invalidating prior labeling and traceability records for clinical settings.

##### Sterilization Protocols for 3D-Printed Radiation Devices

All 3D-printed radiological devices fabricated from a variety of polymeric and composite materials need to be sterilized for clinical implementation [204]. However, sterilization protocols may alter the dimensions and radiological properties of these materials. Thus, material-specific sterilization protocols need to be established for optimal clinical usage [205]. Told et al. [206] have conducted detailed studies on the sterilization of thermoplastic polymers and resin materials for 3D printing of medical devices. Surface disinfection methods and sterilization protocols used in clinical settings that include ethanol treatment, chlorine soaking, gas plasma sterilization, autoclaving, and dry heat sterilization, evaluated for ABS, PETG, PLA, polyamide, white resin, and dental resin materials, respectively. PLA, ABS, and PETG materials should not be sterilized using autoclave or dry heat due to their lower melting points and subsequent physical deformation [207]. However, surface disinfection methods such as ethanol, chlorine soaking, and gas plasma sterilization are recommended, with special care for hollow structures. Multiple cycles of sterilization had no significant effect on the tensile and flexural strength of ABS devices, and thus it was a recommended material for clinical radiological applications [208]. ABS material can also withstand multiple rounds of ionizing radiation without noticeable changes in chemical structure or thermal and mechanical properties [209]. PLA material, when treated with multiple sterilization cycles, displayed lower mechanical strength due to material degradation, and thus should be used for one-off dosing materials. However, blending PLA as a composite with higher melting thermoplastics is a viable option due to its high biocompatibility. Polyamide displayed sustained mechanical and structural stability and is an excellent material for rapid prototyping of radiation devices based on scanning electron microscopy structural analysis [210]. PETG withstood all sterilization and disinfection protocols, and heat-based sterilization was not recommended [211]. All polymeric materials discussed herein displayed good biocompatibility and minimal cytotoxicity with A549 cells after 48 h of incubation [206].

##### Cross-Institutional Reproducibility

Reproducibility of 3D-printed radiological devices across hospitals [212] is limited by heterogeneity in (i) hardware and firmware; (ii) software versions and mesh repair settings; (iii) build orientation/support strategies; (iv) operator training; (v) environmental controls; and (vi) sterilization/post-processing. FDA notes the centrality of validating “worst-case” builds and maintaining design/production records. ASTM F42 [213,214] highlights the need for modality-specific acceptance tests and medical device process validation practices, but coverage for polymers and PoC printing is still evolving. Hospital quality assurance studies document that segmentation steps alone can introduce relative dimensional error before printing, so end-to-end standardization is required for multi-site comparability [215]. As a clinical case study, in a multi-center trial of patient-specific drill guides, one site used SLA and steam sterilization, whereas another used DLP and hydrogen-peroxide sterilization [216]. Despite identical STL files, the second site reported deviation in 3D-printed part geometry after autoclave, with differences consistent with the literature on printer/sterilization interactions.

Thus, specific measures need to be adopted via standardized protocols and verification tests to ascertain that 3D-printed radiation devices can be safely implemented in clinical settings.

## 6. Future Directions

The scope of this review examines the outlook beyond current applications of 3D printing in radiation therapy to explore its transformative potential in shaping next-generation oncology practices. Specifically, advancements in additive manufacturing promise greater personalization, sustainability, and workflow efficiency, while artificial intelligence introduces opportunities for automated design, adaptive planning, and real-time quality assurance. This section addresses future prospects that will shape clinical acceptance and innovation by looking at emerging technology and regulatory frameworks. Advanced regulatory frameworks for 3D-printed medical equipment and AI-driven advancements in treatment planning and device design are two convergent technology frontiers that are shaping the future of radiation therapy. Regulatory organizations like the FDA are developing rules to guarantee safety, reproducibility, and compliance, while allowing for rapid innovation as additive manufacturing becomes essential to the production of patient-specific boluses, immobilization aids, and complicated collimators. Artificial intelligence is simultaneously revolutionizing clinical procedures by providing previously unheard of accuracy and efficiency through automated segmentation, adaptive planning, and generative design. All of these advancements point to a paradigm of change in the direction of intelligent, patient-specific radiotherapy ecosystems. However, achieving these opportunities also presents difficulties with regard to ethical supervision, data protection, regulatory compliance, and extensive clinical validation. Transforming technical potential into safe, efficient, and globally scalable cancer care solutions will require addressing these obstacles.

### 6.1. FDA Regulatory Landscape for 3D-Printed Radiation Devices

The Federal Drug Administration (FDA’s) future direction involves adopting decentralized non-government reviews, shifting its regulatory focus from product-level evaluations to firm-level assessments, and transitioning scrutiny from pre-market to post-market phases. These changes are particularly relevant for digital health technologies and AI-driven medical devices [217]. To enhance regulatory decision-making and address the complexities of emerging digital health technologies, the FDA is considering integrating real-world clinical evidence [218]. The FDA’s Center for Equipment and Radiological Health, which prioritizes safety and effectiveness, regulates 3D-printed medical equipment [203]. Through its Q-Submission Program, which enables developers to receive input on regulatory expectations, such as device classification, testing procedures, and submission processes, the FDA promotes early engagement to enhance clinical translation. This is especially useful for new 3D-printed radiation therapy equipment that might be used in both traditional and digital health settings [219]. The FDA regulates 3D-printed medical devices used for radiation therapy under similar pathways to traditional medical devices, classified by risk level (Class I, II, or III). Based on the device classification and its potential risk, the required level of premarket review (510 (k) submission or Premarket Approval (PMA)) is determined. The FDA has published the document titled “Technical Considerations for Additive Manufactured Medical Devices” specifically to address the AM-related challenges [192]. For radiation therapy applications, where accuracy and repeatability have a direct impact on treatment results, this guidance highlights important factors including build orientation, post-processing, and validation of mechanical and dosimetric features. The guidance provides recommendations for design and manufacturing processes, device testing, and characterization. Lately, 3D-printed devices are being used in radiation therapy applications to provide personalized treatment with enhanced patient comfort. Some of the examples of these devices include patient-specific boluses, personalized immobilization devices, phantoms, customized brachytherapy applicators, and molds. In this rapidly developing field, stakeholders should apply the technological considerations for 3D printing, make sure that all general FDA device laws are followed, and keep an eye out for new, more focused restrictions [192,220,221]. To guarantee consistent device performance and regulatory certification, developers must also guarantee adherence to ISO 13485-certified quality management systems and Good Manufacturing Practices (GMPs) [222]. This approach helps ensure patient safety while allowing regulations to keep pace with the rapidly evolving healthcare landscape. As a result, medical device regulations have adapted and will continue to evolve alongside digital health advancements [218]. Because 3D-printed devices can be customized, there are regulatory issues that require a strong design control model in order to receive FDA approval [223]. To show compliance with the FDA’s Quality System Regulation (21 CFR Part 820), design control documentation should include risk analysis, verification and validation procedures, and traceability matrices [224,225]. However, regulatory frameworks must remain dynamic to address new challenges presented by these technologies [218]. While the FDA’s guideline is crucial for guaranteeing the efficacy and safety of radiation devices made using 3D printing, some contend that the regulatory environment may impede innovation and the quick uptake of innovative technology in clinical settings [226]. Regarding 3D printing at the point of care (clinics or hospitals), the FDA released an unofficial discussion paper. The discussion paper draws attention to regulatory issues and offers possible frameworks for managing devices that are printed onsite while requesting feedback from stakeholders to build formal policy [227,228].

Additionally, the successful development of customized medical devices and patient-specific tissue constructs with 3D printing processes requires careful consideration of several critical factors, including printing parameters, material consistency, biocompatibility, sterility, and mechanical properties [229]. Recent reviews of polymer-based 3D printing highlight significant progress in optimized printing hardware, material development (e.g., thermally conductive, biocompatible filaments), and functional composite applications relevant to biomedicine and radiation therapy [166]. Generally, the 3D-printed medical devices are regulated under the 510 (k) premarket notification pathways. If any novel materials are introduced while 3D printing, then there is a need for Premarket Approval (PMA). Moreover, good manufacturing practices and ISO 13485 quality management systems [228] are very crucial for clinical deployment. Long-term safety and efficacy must be monitored through post-market surveillance methods, such as complaint management, adverse event reporting (21 CFR 803), and device monitoring (21 CFR 821), particularly for devices used in high-risk oncology settings [230,231,232]. For manufacturers to satisfy regulatory requirements, consistent material qualities and ideal printing settings must be guaranteed [223]. In order to comply with FDA regulations, establishing a 3D printing service in radiotherapy necessitates extensive training and multidisciplinary team collaboration [233]. To guarantee clinical integrity and regulatory compliance throughout the device’s lifecycle, institutions should also put internal quality assurance procedures into place, such as phantom-based dosage verification and recurring audits [234]. The layer-by-layer nature of 3D printing plays a crucial role in medical device manufacturing, as material properties and printing parameters directly influence outcomes [229]. For 3D printing equipment, the FDA stresses the value of quality management systems, which include documented risk assessments and maintenance procedures [176]. Nonetheless, challenges such as batch-to-batch consistency issues, material recycling difficulties, and the limitations of different 3D printing technologies remain significant hurdles [229].

### 6.2. Artificial Intellegence (AI)-Driven Radiation Therapy Applications

AI is rapidly transforming radiation therapy planning, automating tasks traditionally performed by radiation therapists (RTs). This shift requires that RTs adapt to these technological advancements and reconsider their roles within interdisciplinary teams. RTs have voiced concerns about AI’s impact on their profession, particularly regarding its effects on multidisciplinary collaboration [235]. AI plays a crucial role in radiation oncology, contributing to prognostic assessment, treatment planning, tumor segmentation, and quality control. It enhances productivity by reducing planning time and promoting standardization, even in resource-limited environments. Advanced AI techniques including deep learning and knowledge-based planning used to optimize treatment processes, highlight AI’s ability to standardize radiation therapy globally by improving tumor segmentation and personalized treatment planning. However, challenges remain, such as the need for extensive data collection and the difficulty of correlating treatment plans with patient outcomes [236].

Patient data privacy is crucial when using imaging data for AI-driven device design. To adhere to ethical and regulatory requirements, sensitive health information must be safeguarded through strong anonymization and secure data processing procedures. Additionally, clinician supervision is necessary to verify results and preserve patient safety, and AI models should be explicable to provide transparency in decision-making. By taking these steps, algorithmic bias risks are reduced, and confidence in AI-assisted healthcare applications is increased [237,238].

One significant development in AI-driven radiation therapy is the Deep Learning On-Demand Assistant, a fully automated, end-to-end clinical platform. The study “Prospective deployment of an automated implementation solution for artificial intelligence translation to clinical radiation oncology” explores the creation and integration of this system. Incorporating an automated model-training pipeline, auto-segmentation, and QA reporting, this approach facilitates AI implementation across various disease sites. It represents a major step toward a universally applicable AI framework, enhancing efficiency and reliability in radiotherapy. Future advancements include improved tumor diagnosis, segmentation, and individualized treatment planning based on comprehensive data analysis and real-time adaptive therapy [239].

AI also enhances treatment uniformity, reduces workload, and streamlines workflow in radiotherapy. Current AI applications include predictive models that integrate clinical and omic data for personalized oncology approaches. Techniques such as three-dimensional dose distribution, reverse planning, and AI-driven strategies have led to increased productivity, reduced workload, and greater treatment consistency. Additionally, predictive AI tools help tailor oncologic strategies, dosage recommendations, and make suggestions. However, these tools require transparency to support clinical decision-making and necessitate extensive validation across large patient populations [240,241].

Automation and AI are significantly improving radiation therapy treatment planning by increasing efficiency and optimizing workflow. RTs remain essential in implementing these technologies, ensuring patient safety and maintaining oversight in clinical practice. Key concerns include safeguarding safe advancements in practice and preventing AI from undermining innovation, oversight, and clinical creativity. Further research is required to assess AI’s impact on professional responsibilities and determine best practices for its integration [242]. In interventional radiotherapy, AI enhances treatment planning by optimizing processes, personalizing treatments, and improving patient outcomes. Despite its advantages, challenges remain, including the need for clinical validation and robust QA protocols. A systematic review of 78 studies published between 2002 and 2024 examined AI-driven approaches for treatment planning, contouring, and outcome prediction. Results demonstrated improved contouring, enhanced planning efficiency, and more accurate prediction. However, integrating AI into clinical practice still requires ongoing validation and the refinement of QA measures [243]. Applying aviation principles to radiotherapy planning, automation increases accuracy and efficiency. However, human oversight remains crucial to prevent risks such as complacency, over-reliance, and attention tunneling. Research highlights the importance of human supervision in complex clinical scenarios. Recommendations include strategies to mitigate automation risks and a proposed taxonomy for automation levels in radiation therapy. Identified research gaps include the need for proactive event learning and further human factors engineering research in radiation oncology [244].

AI algorithms have shown potential in improving tumor motion tracking accuracy compared to traditional methods. This is particularly relevant for managing intrafraction motion during radiation therapy. However, challenges such as data bias, transparency issues, and processing speed must be addressed. AI has demonstrated improved accuracy in tumor tracking, yet its effectiveness is limited by biases in data collection, restricted algorithm generalization, and the lack of standardized AI implementation in clinical practice [245].

AI algorithms can optimize 3D-printed radiation devices using generative design techniques, which enable complicated geometries that are not possible with conventional methods [246]. AI algorithms enable the development of highly personalized medical devices that are adapted to the unique anatomy of each patient, improving the efficacy of therapies [247]. The integration of AI with 3D printing has the potential to transform healthcare by automating processes and minimizing errors [248]. With AI real-time process monitoring, the 3D printing process can be controlled, which minimizes errors and guarantees high-quality results. By automatically modifying printing conditions in response to feedback, closed-loop systems can be used to improve manufacturing process reliability [249]. Three-dimensional printing for medical devices is made more efficient by AI, which improves material selection, guarantees component quality, and enables customized modifications. By using recycling and organic resources, it enables increased production efficiency, better functionality, and environmental advantages [250,251]. By optimizing material utilization, AI improves 3D printing and can reduce waste by up to 30% [251]. Medical devices can be made available on time by using machine learning approaches to optimize production schedules and supply chain management [252]. AI can improve safety standards, maintain regulatory compliance, and streamline design procedures for 3D-printed medical devices. In order to improve patient care and device functionality, it interfaces with the Internet of Medical Things and makes customized treatment solutions easier [253]. For the advancement of training and education, clinical training results are enhanced by an AI-driven 3D-printed model, which gives medical personnel realistic training tools [251]. Because simulation models may mimic different medical circumstances, people can be better prepared for actual situations [254].

In summary, the wider adoption of AI and advanced 3D printing technologies with multiple materials in the radiation therapy application enables customized, adaptive, and automated treatment planning with minimal toxins and improved patient safety. However, successful integration requires overcoming challenges related to data quality, clinical validation, and maintaining human oversight. As AI continues to evolve, radiation therapists must adapt to these changes while ensuring that technological advancements align with patient safety and treatment efficacy.

## 7. Conclusions

Three-dimensional printing technologies enable a high degree of customization, enabling patient-specific devices that conform accurately to patients’ personalized anatomy and therapeutic needs. The integration of radiation therapy with 3D printing technology represents a significant advancement in medical device design, manufacturing, and clinical applications. This review delves into the comparative advantages, such as design flexibility, sustainability, production time, and cost, compared to the traditional processes for radiation medical devices. Three-dimensional printing has demonstrated promising advancements for developing medical devices that enhance dose distribution, optimize the comfort of patients, and minimize the exposure of healthy tissues to photon, electron, and proton radiation therapies. A specialized case study on 3D-printed spatially fractionated radiation therapy—the GRID collimator—and advancements in personalized radiation dose modulation were presented. Despite these advances in the 3D printing of radiation therapy medical devices, challenges prevail, including standardized workflows for designing and manufacturing 3D-printed devices, regulations, and the adoption of large-scale clinical studies. Variability in 3D-printed device performance, operator expertise, and infrastructure requirements could limit scalability, particularly in resource-constrained settings. Regulatory pathways and ethical considerations surrounding patient selection criteria further underscore the need for comprehensive guidelines informed by large-scale, multi-center trials. AI-driven device design, dose optimization, automated quality verification, and validation with real-time monitoring of treatment can advance radiation therapy medical devices based on the specific patient requirements. This review also provided an overview of the evolving regulatory landscape, specifically the FDA guidelines for radiation medical devices in healthcare. While the findings presented offer promising insights into the potential clinical utility of this approach, limited demographic diversity, methodological variability including differences in protocols, outcome measures, and follow-up durations need further investigation. Thus, establishing guidelines for validation, safety, and clinical implementation are paramount to ensure reliability, reproducibility, and patient safety for broad clinical adoption. Overall, this review study serves as a guideline for researchers, biomedical engineers, clinicians, industry stakeholders, regulatory bodies, and manufacturers to evaluate 3D-printed radiation devices for customized patient treatment.

## Figures and Tables

**Table 1:** Cancer therapeutic modalities comparison: techniques, applications, advantages, and limitations.

Therapy Type	Technique	Applications	Advantages	Limitations/Adverse Actions
Surgery	Physical removal of tumors or affected tissues	Solid tumors, early-stage cancers, symptom relief	Potentially curative, localized treatment	Risk to nearby healthy organs, long recovery, bleeding, infection, not suitable for blood cancers
Chemotherapy	Chemical agents disrupt cell division and DNA/RNA synthesis	Leukemia, lymphoma, sarcoma, multiple myeloma	Systemic treatment, can shrink tumors, used in combination therapies	Toxicity, affects healthy cells, side effects vary by drug class
Immunotherapy	Stimulates or modifies the immune system to target cancer cells	Melanoma, lung cancer, prostate and breast cancer (via hormone therapy)	Utilizes the body’s natural defenses, long-term response potential	Immune-related adverse effects, limited efficacy in some cancers
Targeted Therapy	Targets specific molecules involved in cancer growth (e.g., monoclonal antibodies)	Leukemia, lymphoma, colorectal, lung, pancreatic, breast cancers	Precision treatment, fewer side effects compared to chemotherapy	Unique toxicities (e.g., cardiac dysfunction, rash), resistance may develop
Radiation Therapy	Uses ionizing radiation to damage DNA of cancer cells	Solid tumors, symptom relief, pre/post-surgery treatment	Localized treatment, effective in many cancers, 3D printing enhances precision	Can harm nearby healthy tissue, delayed cell death, side effects depend on dose and location

**Table 2:** Commercial v/s customized GRID parameter comparison.

Manufacturer	Type	Size	Weight	Dimension	Material
RPD (Albertville, MN, USA)	GRID Photon Block, Varian Type III (with MLC), 65.4 cm	7.5 cm	48 lbs.	~10″ × 10″; 8″–10″ squared…tray is ~1.5″ and depends on tray and distance of MLC	Low melting alloy (Cerrobend)
.decimal, Inc. (Sanford, FL, USA)	GRID therapy (SFGRT)—photon therapy product	7.5 cm	39.62 lbs.	7.133 in (x) × by 6.757 in (y)	Brass
3D-printed GRID (Greensboro, NC, USA)	GRID therapy—photon therapy	7.5 cm	1.39 lbs.	29.5 cm × 29.5 cm	Acrylonitrile butadiene styrene (ABS)

**Table 3:** Performance comparison of 3D-printed radiation therapy medical devices.

Medical Device	Material	Beam Energy	Measurement Instrument	Gamma Criteria	Dosimetry Parameters	Sample Size	Geometric Accuracy	Cost-Efficiency	Use Case	References
Implant	PEEK-Biocompatible Polymer	6 MV photon	Matrixx FFF system	3%/3 mm	HU Value = ±3% Dose Difference < 2% Gamma Pass Rate > 96%	5	High accuracy compared to commercial flat bolus	High cost-efficiency–low artifact	Best for radiation therapy treatment	Yang et al. [104]
Implant	Titanium Mesh	6 MV photon	Matrixx FFF system	3%/3 mm	Moderate HU distortion Dose Difference < 3% Gamma Pass Rate > 97.4%	5	Highest accuracy with minimal air gaps	Moderate cost-efficiency, lower artifact than solid titanium	Preferred if titanium is required; suitable for post-operative radiotherapy	Yang et al. [104]
Implant	Solid Titanium	6 MV photon	Matrixx FFF system	3%/3 mm	Severe HU distortion Dose Difference < 3% Gamma Pass Rate ~89%	5	Lowest accuracy but good surface finish	Low cost-efficiency requires additional correction, risk of dose uncertainty	Not recommended for radiotherapy patients; causes inaccurate dose calculation	Yang et al. [104]
Immobilization	PLA-based	6 MV X-rays in flattening filter	Anthropomorphic phantom and a Gafchromic EBT3 film	3%/3 mm	HU ≈ tissue DVH changes up to 10% Gamma Pass Rate = 92.14%	49	Less accurate but still within clinical tolerances	Moderate: increases skin toxicity risk requiring mitigation but improves immobilization and reproducibility	Used for improved patient positioning; requires TPS inclusion to avoid skin dose underestimation	Yin et al. [105]
Bolus	Premium PLA	Photon/Electron	CT scan (Philips Brilliance 16 slice wide bore)	Not reported	HU Value = 80 ± 8 Quality Assurance verified Gamma Pass Rate not reported	2089	High reproducibility with thickness tolerance ±1 mm (photons), ±0.5 mm (electrons	High cost-efficiency: low material waste	Custom bolus for head and neck, breast and skin cancer radiotherapy	Basaula et al. [71]
Breast Bolus	PLA + Gel	6 MV photon	MOSFET dosimeters	Not reported	HU ≈ tissue Dose difference = 2.7% Gamma Pass Rate Not Reported	7	Enhanced accuracy with improved fit vs. convention bolus	High efficiency due to repeatability	Increases dose precision at skin and shallow depths; reduces cold spots from poor contact	Takanen et al. [61]
Photon Block	PLA shell + tungsten BBs	6 MV photon	Ionization chambers and TrueBeam MV imaging	Not reported	HU not reported Dose Difference = 2% Megavoltage image profile agreement within 1%; Jaccard similarity better than Cerrobend	3	Excellent geometric accuracy with robust mechanical stability	Low-cost PLA shell (minimal mass) and reusable tungsten ball bearings	Enables patient-specific shielding (mantle fields, pelvis, lung, ovary shielding)	Schulz [54]
Bolus	PLA/TPU	6 MV, 10 MV photon	Advanced Markus ionization chamber	Not reported	HU ≈ tissue PDD difference < 3% Gamma Pass Rate not reported	Not reported	Thickness accuracy within 0.1 mm/mm	High efficiency with minimal materials cost and consumables	Suitable for standard anatomical areas, less comfortable but stable fit	Zhang et al. [63]
Bolus	Silicone Rubber	6 MV photon	FG65-P detector, Gafchromic EBT3 film	3%/2 mm	Similar to commercial bolus Dose Difference = 1.1% Gamma Pass Rate = 93.9%	3	Fit: perfect conformity to irregular surfaces (nose, cheek, neck) reducing air gaps	Up to 15× cheaper than commercial bolus	Best for head and neck regions with irregular contours	Chatchumnan et al. [106]
Phantom + Bolus	PLA	6 MeV photon	Gafchromic film	Not reported	HU = −32 Dose increased 26–52% vs. no bolus Gamma Pass Rate not reported	5	Accurately fits on cheeks, nose, scalp with minimal air gaps	Low-cost, recyclable PLA and in vitro quality assurance	Used for dose build-up in superficial head and neck cancers; improves surface dose coverage	Jreije et al. [107]
Bolus	PLA, ABS, PET-G	Not reported	Not reported	Not reported	HU range: −144 ± 9 Dose 89–93% Gamma Pass Rate not reported	Not reported	High accuracy with better geometric precision	Low-cost polymers; printing inexpensive; comparable to gel bolus	Optimal 3D bolus materials; selection of Premium PLA, Standard PLA, PET-G suitable for clinical bolus	Ciobanu et al. [108]
Phantom	PLA, ABS	Varies	Varies	Varies	HU range: 500–1000 Quality Assurance feasible Gamma Pass Rate not reported	Varies	High accuracy with perfect filling and negligible transmission	Moderately cost-effective with minimal labor and streamlined workflow	Custom electron shields for skin cancer	Tino et al. [83]
Bolus	Various polymers	Not reported	CT Big Bore, HandySCAN™ 300	Not reported	HU improved with scanner fit Better Conformity Gamma Pass Rate not reported	10	Superior accuracy with smoother shape, better anatomical conformity	Highly cost-effective with minimal material cost and reduced workflow time	Ideal for head and neck, breast, hand, ear, and complex geometries	Dipasquale et al. [109]
Bolus	TPU	Varies	Varies	Not reported	HU ≈ tissue Improved surface dose compared to commercial bolus Gamma Pass Rate not reported	Varies	Accurate 1 cm thickness; consistent material density	Inexpensive and similar cost to PLA.	Used in facial, auricle, phantom studies; alternative to PLA with similar performance	Wang et al. [110]
Breast Bolus	PLA	10 MV photon	MOSFET dosimeter	Not reported	HU Not reported Dose −0.6% to −1.1%. Gamma Pass Rate not reported	1	Fit accuracy: No air gaps for 200–300 cc breasts	Highly cost-effective with reusable digital design and no manual shaping	Ensures high surface dose when treating whole-breast irradiation with tangential fields	Park et al. [52]
Bolus	Ninjaflex/Wolfbend	Photon/electron	PTW Roos and Advanced Marcus ionization chamber	Not reported	±15 HU/±5 Dose < 2% Gamma Pass Rate > 95%	Not reported	Geometric precision with improved nose fit vs. standard bolus	Inexpensive process with reproducible prints	Ideal for irregular surfaces—nose, vulva, breast edges; eliminates air gaps and improves surface dose consistency	Malone et al. [111]
Patient-specific Bolus	ABS, PLA	6 MV photon	Gafchromic EBT2 film	5%/2 mm, and 5%/3 mm respectively	HU~260 Validated by Film Gamma Pass Rate = 86.5% /95% respectively	1	Perfectly reproduces anatomical curvature	Highly cost-effective with minimal materials and printer expenses	Highly conformal bolus for nose, scalp, irregular head and nose surfaces	Burleson et al. [59]
Electron Bolus and Proton Compensator	PLA (bolus), Polyamide (compensator)	Electron/proton	Bragg peak ionization chamber	Not reported	Bolus: 106.5 ± 15.2; Compensator: −70.1 ± 8.1 Dose not reported	4	Higher geometric fidelity	Minimal cost with better uniformity leading to fewer QA failures compared to milling	Ideal for proton double-scattering therapy, e.g., prostate therapy	Zou et al. [65]
Customized Bolus	PLA	6 MV photon	Exradin A19 ionchamber, SuperMAX electrometer, Gafchromic EBT2 film	Not reported	Not reported Good agreement with TPS; qualitative dose escalation not reported	1	Excellent surface conformity on irregular anatomy	Low-cost with minimal post-processing	Ideal for nose, ear, scalp, and other irregular head and neck surfaces where flat bolus fails	Kim et al. [53]

Note: Abbreviations: PLA; ABS; PEEK (polyetheretherketone); DVH (dose-volume histogram); BBs (ball bearings); PET-G (polyethylene terephthalate glycol, clear durable thermoplastic); TPU (thermoplastic polyurethane, a flexible, durable polymer); TPS; PDD.

## Data Availability

No new data was created or analyzed in this study.
